# Supplementary material for: Nova-ST: Nano-patterned ultra-dense platform for spatial transcriptomics
Source: Cell Rep Methods. 2024 Aug 6;4(8):100831. doi: 10.1016/j.crmeth.2024.100831 (PMC11384075; doi:10.1016/j.crmeth.2024.100831)
Supplement: Document S1. Figures S1–S5 and Table S1 [file mmc1.pdf]

Cell Reports Methods, Volume *4*

Supplemental information

Nova-ST: Nano-patterned ultra-dense platform for spatial transcriptomics

Suresh Poovathingal, Kristofer Davie, Lars E. Borm, Roel Vandepoel, Nicolas Poulvellarie, Annelien Verfaillie, Nikky Corthout, and Stein Aerts

Supplementary Figures:

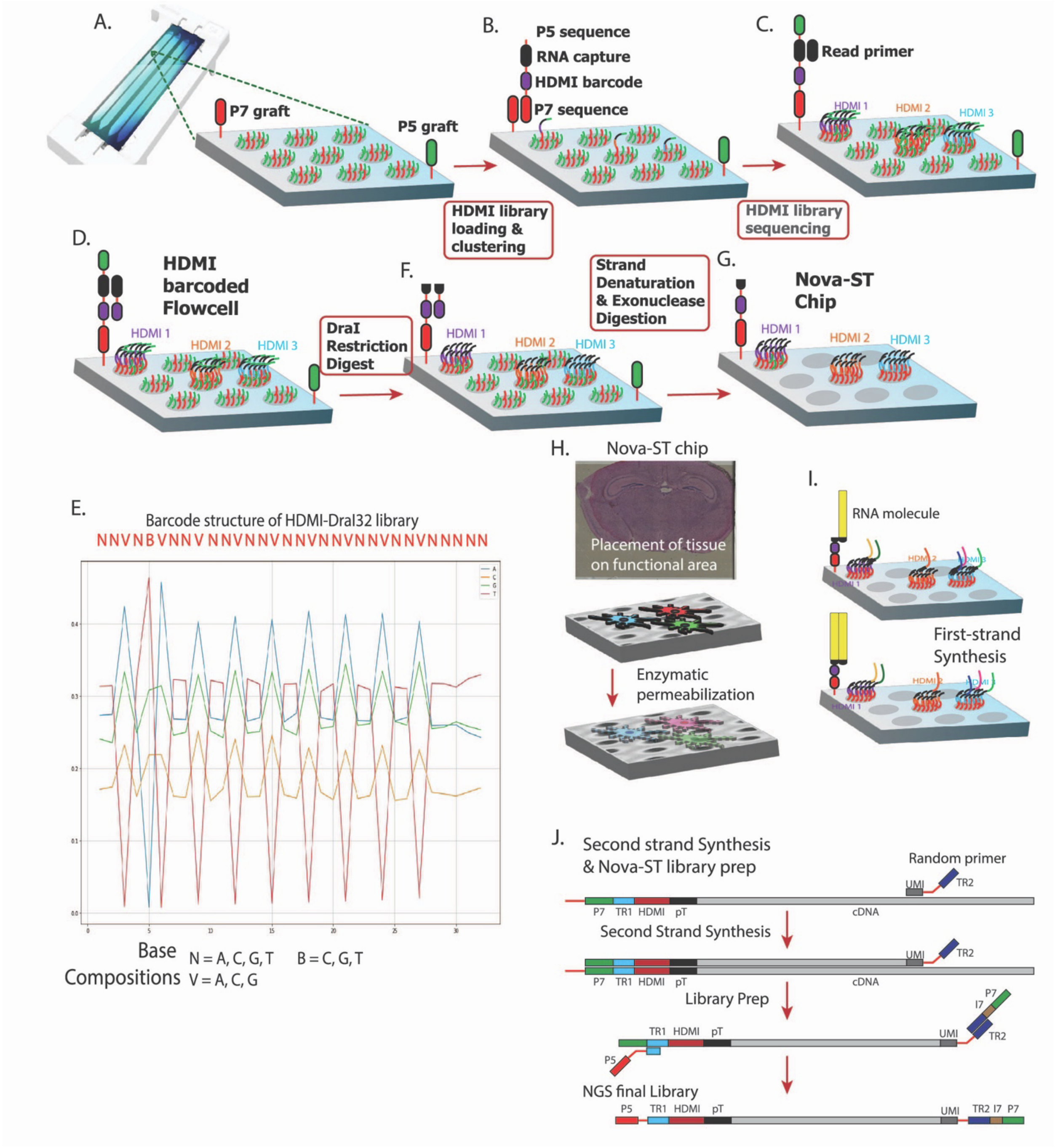

Figure S1: Summary of Nova-ST workflow. Related to Figure 1, 2 & STAR Methods section.
A.) Cartoon depicting nanowells with the functionalized sequencing handles in the NovaSeq 6000 S4 flow cell.
B-D.) HDMI sequencing workflow where the HDMI oligonucleotide (HDMI-DraI 32) undergoes annealing with the sequencing handles in the nanowell followed by isothermal bridge amplification, in which several thousand copies of the HDMI strand are created. During sequencing, a custom read primer (Read1-DraI primer), reads the HDMI spatial barcode in the backbone of the HDMI oligonucleotide sequence.

E.) An example of base composition of the sequenced HDMI library compared to the actual sequence of the HDMI spatial barcodes in the HDMI-DraI32 ultramer.
F.) Post sequencing, the Dra-I restriction enzyme cuts the double strand sequence at the TTTAAA locus to free the capture domain.
G.) The double stranded DNA is denatured using caustic treatment to expose the RNA capture domain for spatial transcriptomics.
H.) Tissue overlayed on the functional surface of the Nova-ST chip. Diagrammatic description of the enzymatic digestion of tissue on the surface of the Nova-ST chip.
I.) mRNA captured from the permeabilized tissue followed by the first strand synthesis on the surface of the Nova-ST chip.
J.) Downstream processing of the spatial library after the removal of the tissue from surface of Nova-ST chip, where second strand synthesis is achieved by a random primer extension (RPE). The extended second strand product is denatured and the rest of the RPE product amplification and the indexed NGS library preparation is performed in a tube.

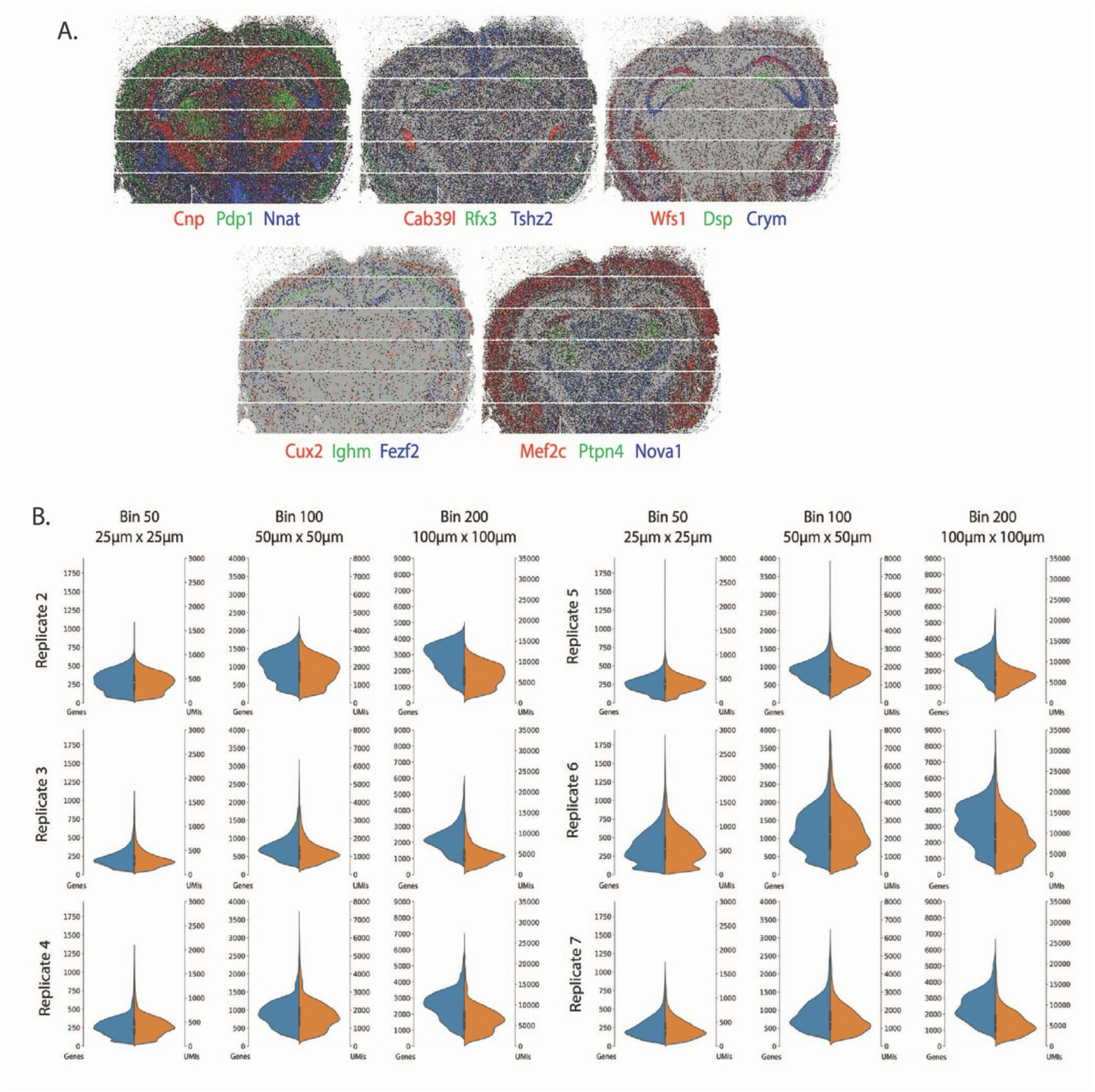

Figure S2: Quality comparisons of Nova-ST samples showing spatial localizations and general sequencing statistics. Related to Figure 3.

A.) Various sets of spatially expressed genes displaying specific patterns throughout the brain including specificity in the cortex and hippocampus.

B.) Gene (blue) and UMI (orange) distributions for all shallowly sequenced replicates at all three bin sizes (50, 100 and 200).

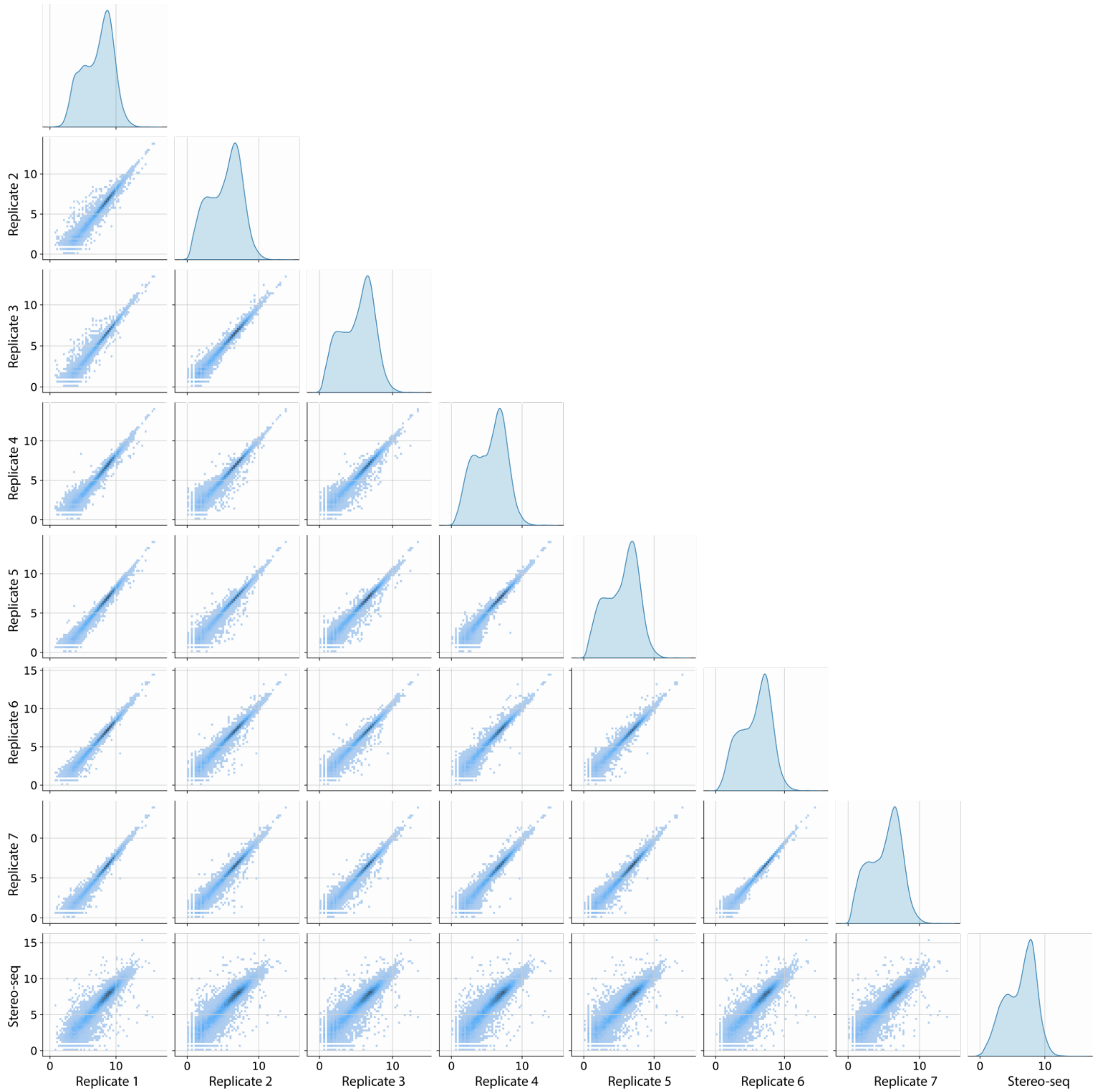

Figure S3: Correlation analysis with all replicates in Nova-ST trials. Related to Figure 3.
Pairwise comparisons of summed gene counts within each sample, log-log axes. Darker areas indicate higher genes density. Self-comparisons are excluded, only genes found in all samples are included.

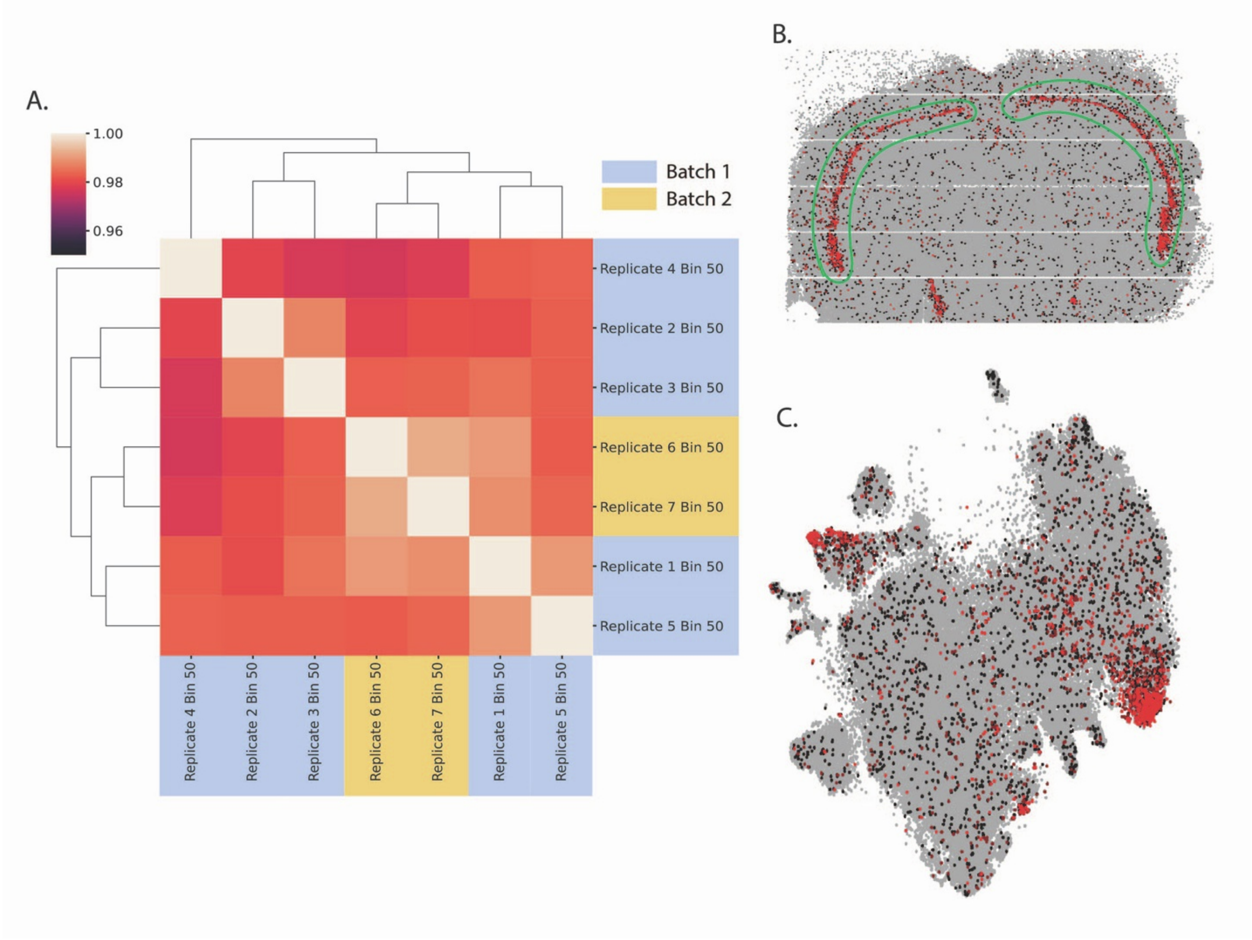

Figure S4: Summed genes correlation analysis with replicates in Nova-ST trials and diffusion of transcripts with Nova-ST. Related to Figure 3.

A.) Pairwise Pearson correlation values of summed gene counts (log scale) between each of the replicate samples. Hierarchical clustering shows no batch effect between different batches of Nova-ST chips sample.

B-C.) Spatial visualizations and t-SNE for Nova-ST DS data. Expression of Ccn2 is localized to the cortex layer 6b and corresponding location of these bins in t-SNE is highlighted by the green contours.

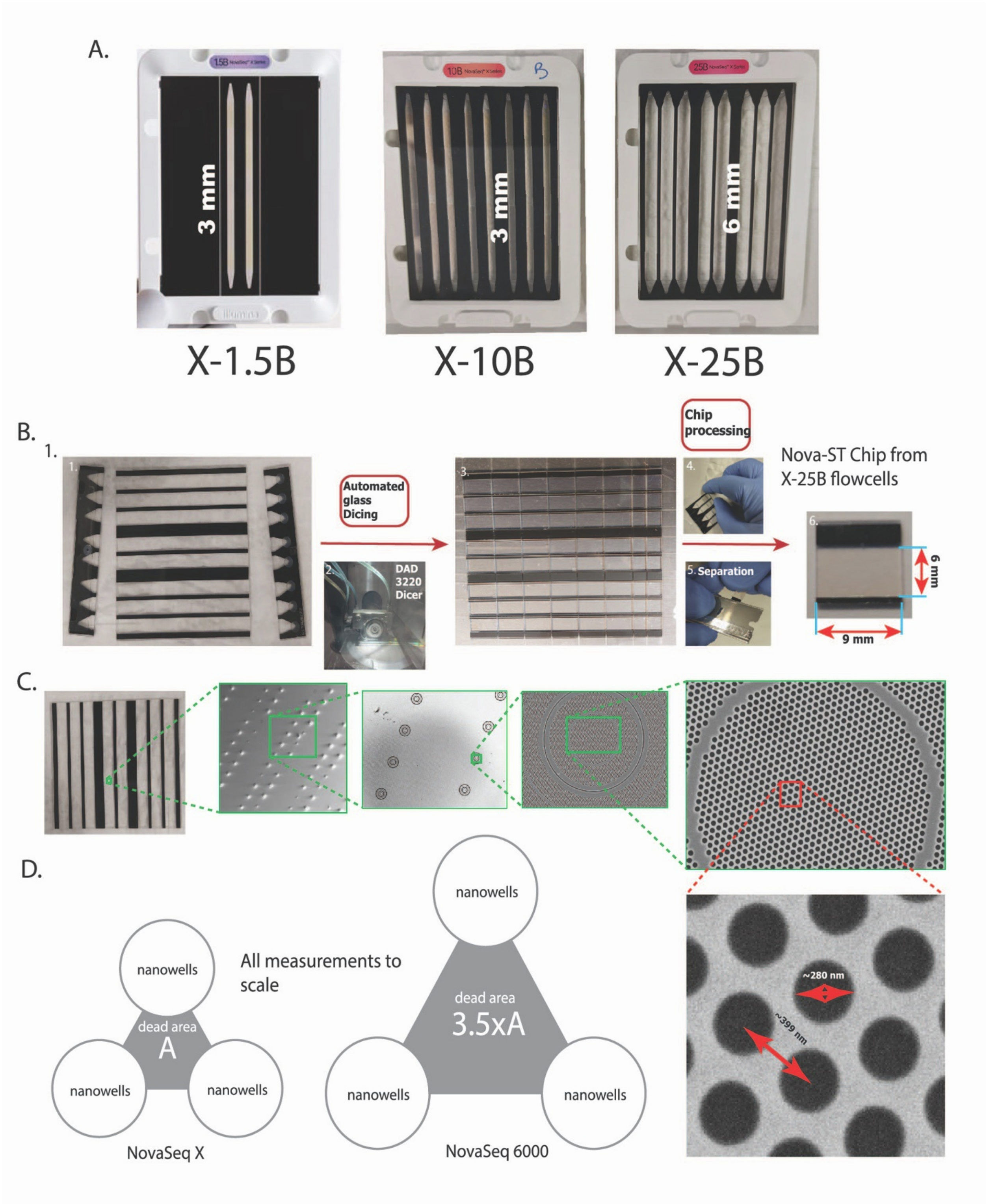

Figure S5: NovaSeq-X processing details for preparing Nova-ST chips. Related to Figure 1 & STAR Methods.

A.) Different flow cell variants of the NovaSeq X sequencer, with the thickness of the flow channel (active area) indicated.

B.) Details of automated cutting: 1. A grid pattern of chip size is created, along which the flow cells are cut to prepare the Nova-ST chips. 2. Cutting the flow cell into chips using the DAD 3220 Dicer. 3. The diced flow cell, still attached

to a dicing tape. 4. Removal of the chips from the backing. 5. Separating the chip layers using a sharp razor blade 6. Final Nova-ST chips derived from NovaSeq-X flowcell.

C.) Electron micrograph characterization of the functional surface of the X-25B flow cell. Zoomed-in pictures of different features on the functional surface of the flow cell and the dimensions and the pitch separation of nanowells arranged in a hexagonal grid pattern.

D.) Pictorial description of a scaled comparison of the dead space area between the nanowells in the NovaSeq X and 6000 flow cells.

Supplementary Tables

Table S1: Sequencing statistics related to the different replicates of Nova-ST replicates. Related to Figure 3.

	total_reads	valid_hdmis	invalid_hdmis	discarded_hdmis	relevant	not_relevant
Replicate_1	1229694204	815856409	413837043	0	815856409	0
Replicate_2	76787319	50444015	26342673	0	50444015	0
Replicate_3	89431881	56140800	33289381	0	56140800	0
Replicate_4	111141907	71832838	39308771	0	71832838	0
Replicate_5	97427512	63896606	33530653	0	63896606	0
Replicate_6	139751725	94143058	45607905	0	94143058	0
Replicate_7	79238612	51419396	27818647	0	51419396	0
BGI_Mouse_Brain	1229691448	977259390	252432058	10742731	861023071	116236319

	mapped	unmapped	multimap	annotated	unannotated	dedup_umis
Replicate_1	667007549	122433935	25961455	5.71E+08	95759874	208395033
Replicate_2	42562771	6021148	1757650	37920155	4642616	29806967
Replicate_3	45662443	8366885	1970136	39587409	6075034	25218192
Replicate_4	61137185	8304585	2236574	53594687	7542498	34405898
Replicate_5	54293446	7344408	2121376	47846949	6446497	34647069
Replicate_6	74001874	17058971	2876680	61874500	12127374	46641749
Replicate_7	39780826	9906065	1555548	33864218	5916608	25640370
BGI_Mouse_Brain	671363777	53511081	136148213	5.39E+08	132832578	84854065

	dup_umis
Replicate_1	362852642
Replicate_2	8113188
Replicate_3	14369217
Replicate_4	19188789
Replicate_5	13199880
Replicate_6	15232751
Replicate_7	8223848
BGI_Mouse_Brain	453677134
