# Supplementary material for: Nova-ST: Nano-patterned ultra-dense platform for spatial transcriptomics
Source: Cell Rep Methods. 2024 Aug 6;4(8):100831. doi: 10.1016/j.crmeth.2024.100831 (PMC11384075; doi:10.1016/j.crmeth.2024.100831)
Supplement: Document S2. Article plus supplemental information [file mmc2.pdf]

Cell Reports
Methods

Report

Nova-ST: Nano-patterned ultra-dense platform for spatial transcriptomics

Graphical abstract

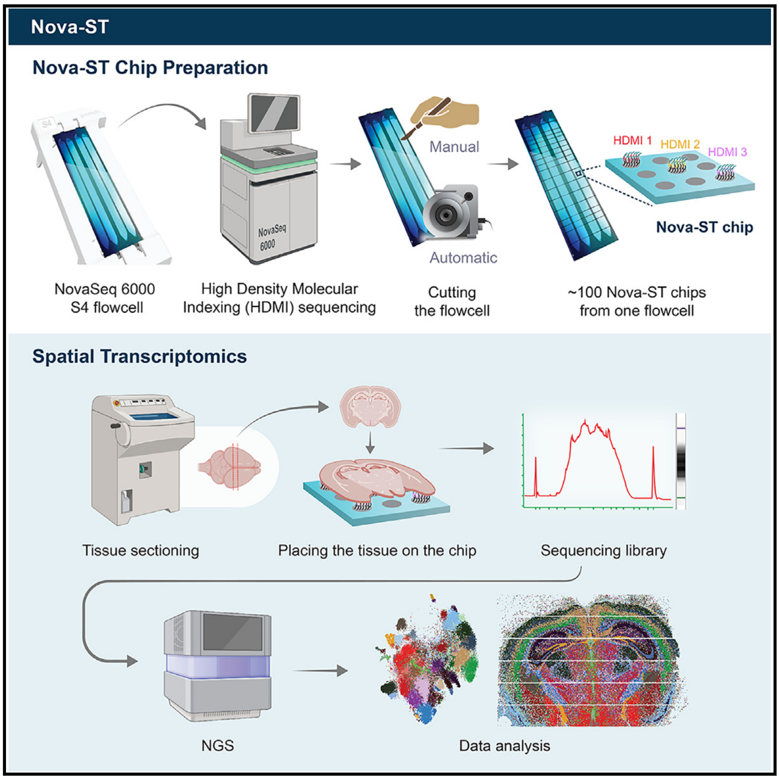

Highlights

- Open-source spatial transcriptomics workflow on Illumina NovaSeq 6000 or X flow cells
- It facilitates low-cost and high-resolution spatial atlasing of large tissue sections
- Nova-ST computational pipeline provides automated data processing
- Nova-ST demonstrates equivalent or enhanced performance to commercial equivalents

Authors

Suresh Poovathingal, Kristofer Davie, Lars E. Borm, ..., Annelien Verfaillie, Nikky Corthout, Stein Aerts

Correspondence

suresh.poovathingal@kuleuven.be (S.P.), stein.aerts@kuleuven.be (S.A.)

In brief

Poovathingal et al. develop Nova-ST, a low-cost, whole-transcriptome spatial workflow based on Illumina NovaSeq flow cells. The nano-patterned structure allows high-resolution, single-cell spatial profiling of large tissue sections from diverse species and tissue types. Nova-ST provides equivalent or superior performance to other comparable technologies at a fractional cost.

Poovathingal et al., 2024, Cell Reports Methods *4*, 100831
August 19, 2024 © 2024 The Author(s). Published by Elsevier Inc.
https://doi.org/10.1016/j.crmeth.2024.100831

CellPress

Cell Reports Methods

CellPress
OPEN ACCESS

Report

Nova-ST: Nano-patterned ultra-dense platform for spatial transcriptomics

Suresh Poovathingal,[1,2,5,10,*] Kristofer Davie,[1,3,10] Lars E. Borm,[1,4,6] Roel Vandepoel,[1,4,6] Nicolas Poulvellarie,[7] Annelien Verfaillie,[8] Nikky Corthout,[9] and Stein Aerts[1,4,5,6,11,*]

[1]VIB Center for AI & Computational Biology (VIB.AI), 3000 Leuven, Belgium
[2]VIB-KU Leuven Center for Brain & Disease Research, CBD Technologies, Single Cell & Microfluidics Expertise Unit, 3000 Leuven, Belgium
[3]VIB-KU Leuven Center for Brain & Disease Research, CBD Technologies, Single Cell Bioinformatics Expertise Unit, 3000 Leuven, Belgium
[4]VIB-KU Leuven Center for Brain & Disease Research, 3000 Leuven, Belgium
[5]Aligning Science Across Parkinson's (ASAP) Collaborative Research Network, Chevy Chase, MD 20815, USA
[6]Department of Human Genetics, KU Leuven, 3000 Leuven, Belgium
[7]VIB-KU Leuven Center for Brain & Disease Research, CBD Technologies, Neuroengineering Expertise Unit, 3000 Leuven, Belgium
[8]Genomics Core, KU Leuven, 3000 Leuven, Belgium
[9]Bio Imaging Core, VIB-KU Leuven Center for Brain & Disease Research, 3000 Leuven, Belgium
[10]These authors contributed equally
[11]Lead contact
*Correspondence: suresh.poovathingal@kuleuven.be (S.P.), stein.aerts@kuleuven.be (S.A.)
https://doi.org/10.1016/j.crmeth.2024.100831

MOTIVATION Spatial transcriptomics analysis is becoming exceedingly important in biomedical and clinical research. Several platforms for spatial transcriptomics are currently available, but most of these technologies are commercialized, inflexible, and cost prohibitive. The motivation for this work was to establish an open-source, flexible, and sensitive sequencing-based spatial transcriptomics platform that can provide a considerable cost advantage for performing large-scale spatial profiling. We provide thorough and in-depth guidance and resources both for the experimental and computational components of the workflow to facilitate easy implementation of Nova-ST.

SUMMARY

Spatial transcriptomics workflows using barcoded capture arrays are commonly used for resolving gene expression in tissues. However, existing techniques are either limited by capture array density or are cost prohibitive for large-scale atlasing. We present Nova-ST, a dense nano-patterned spatial transcriptomics technique derived from randomly barcoded Illumina sequencing flow cells. Nova-ST enables customized, low-cost, flexible, and high-resolution spatial profiling of large tissue sections. Benchmarking on mouse brain sections demonstrates significantly higher sensitivity compared to existing methods at a reduced cost.

INTRODUCTION

Characterizing and modeling complex tissues requires an understanding of the spatial cellular organization and interactions between cells in the context of normal and pathological states.[1,2] Multiplexed *in situ* imaging-based assays measure RNA expression at subcellular resolution, but their implementation is nontrivial and requires pre-selected gene panels for measurement.[3] Compared to *in situ* methods, spatial barcoding methods based on oligonucleotide arrays are straightforward, often easier to implement, and enable unbiased whole-transcriptome analysis. Spatial transcriptomics (currently, 10× Genomics Visium and Visium HD)[4,5] is one such method that is widely used for spatial RNA sequencing. Other sequencing-based spatial assays, such as HDST[1] and Slide-seq (commercialized by Curio Biosciences),[6] use randomly barcoded bead layers for RNA capture[7] or microfluidic technology to perform deterministic spatial barcoding. Most of these methods are, however, limited by low spatial resolution, where the active capture area cannot achieve single-cell resolution.

Recently, several methods based on nano-patterned arrays have emerged that can potentially provide whole-transcriptome capture at subcellular resolution.[8–10] These methods also allow fine-tuning of the size of the capture array to achieve near single-cell resolution. Stereo-seq from BGI STomics uses randomly barcoded DNA nano-balls captured on a nano-patterned array.[8]

© 2024 The Author(s). Published by Elsevier Inc.
This is an open access article under the CC BY license (http://creativecommons.org/licenses/by/4.0/).

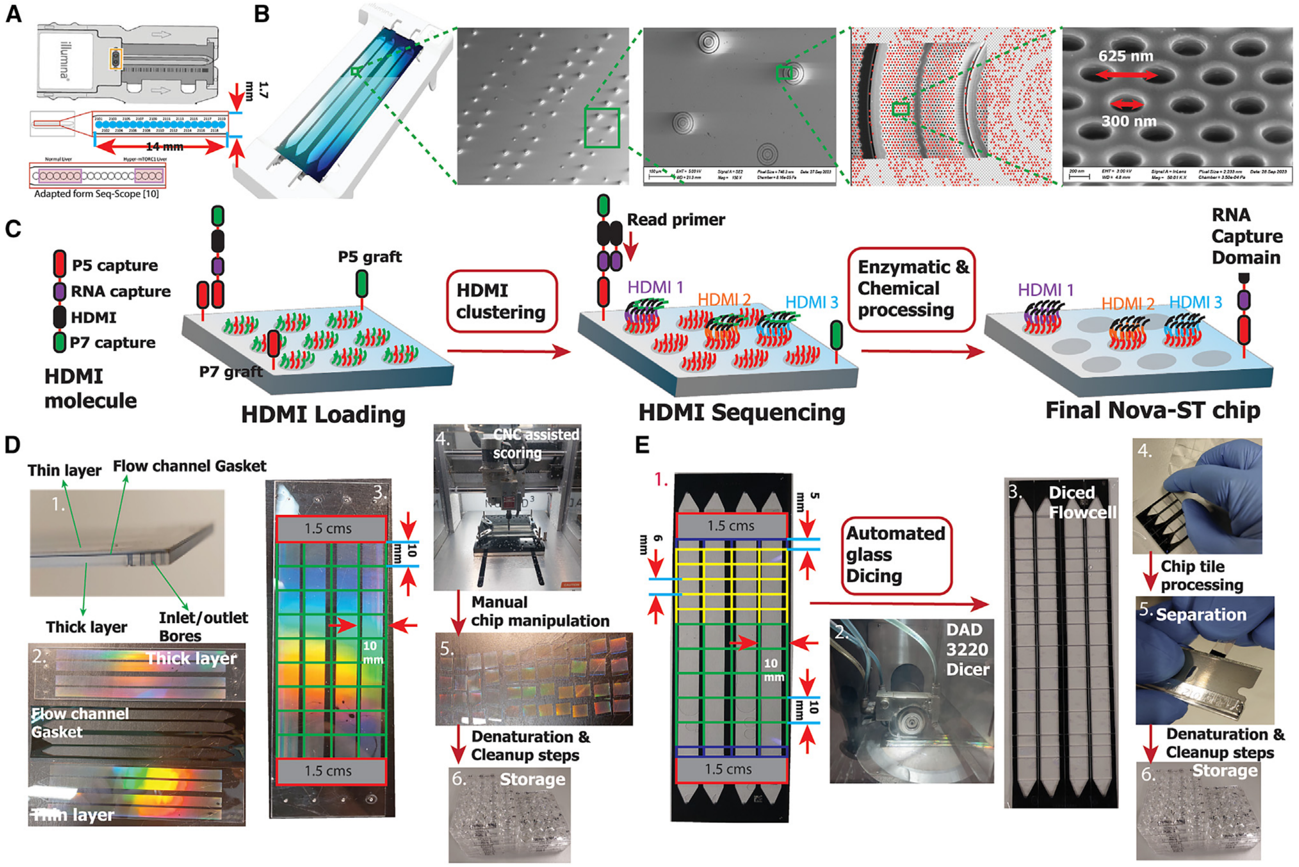

Figure 1. Workflow for Nova-ST chip preparation

(A) Details of MiSeq flow cell used in the Seq-Scope platform.[10] Expanded view of the MiSeq's flow channel indicating the functional area.

(B) Electron microscopy details of the nano-well pattern on the functional surface of the NovaSeq 6000 sequencing flow cell.

(C) Zoomed-in illustration of the nano-wells showing the HDMI sequencing on the NovaSeq 6000 flow cell and the finalized Nova-ST chip with single-stranded DNA having HDMI sequences and RNA capture domains.

(D) Post-processing details of manual cutting: (1) details of the NovaSeq 6000 glass chip showing the thin and thick glass layers sandwiched by a flow gasket. (2) Separated layers of NovaSeq 6000 flow cell exposing the functional surface of the sequencing flow cell. (3) 1 × 1 cm grid, along which the flow cells are cut to prepare the Nova-ST chips. 1.5-cm-thick regions near the entrance and the exit of the flow cell are not sequenced by the NovaSeq 6000, and this region is either discarded or kept for optimizations (gray region). (4) Scribing of the Nova-ST glass layers using the NOMAD 3 CNC milling machine. (5) Final cut 1 × 1 cm Nova-ST chips. (6) Storage of Nova-ST chips in 24-well plates.

(E) Details of automated cutting: (1) a grid pattern of various sizes, along which the flow cells are cut to prepare the Nova-ST chips. (2) Cutting the flow cell into chips using the DAD 3220 Dicer. (3) The diced flow cell still attached to a dicing tape. (4) Removal of the chips from the backing. (5) Separating the thin and thick layers using a sharp razor blade. (6) Storage of Nova-ST chips in 24-well plates.

Alternatively, Seq-Scope repurposes the Illumina MiSeq flow cell to perform spatial RNA sequencing.[10] In the latter method, spatial barcoding is achieved using local bridge amplification of DNA libraries containing random spatial barcodes.[10] In Seq-Scope, MiSeq flow cells have surface functionalization in the contiguous circular pattern in the imaging area (blue numbered circles in <2 mm imaging area in Figure 1A), decreasing the effective spatial footprint and limiting the size of tissues able to be profiled. With a single MiSeq flow cell, it is only possible to perform spatial sequencing of a total of ~10–14 mm^2, meaning that for most practical purposes related to large-scale, high-throughput spatial sequencing of tissues required for cell-atlasing efforts, or for large sectioned clinical samples, Seq-Scope offers very limited possibilities.

In this work, we developed Nova-ST, a cost-effective and easy-to-use spatial transcriptomics platform to perform spatial sequencing on large tissue sections using high-density, patterned flow cells from Illumina. We chose the NovaSeq 6000 S4 flow cells, which provide the largest area for spatial sequencing, amounting to roughly 6,000 mm^2, making spatial profiling cost effective. We further demonstrate the possible adaptation of the Nova-ST workflow to the recently introduced NovaSeq X series of flow cells. Nova-ST consists of both an experimental and a computational workflow. Guidance and resources needed for implementing and performing the Nova-ST experimental workflow are available at https://www.protocols.io[11,12] and https://nova-st.aertslab.org/, while the computational pipeline is available at https://github.com/aertslab/Nova-ST.

RESULTS

Generation of spatial transcriptomics arrays for Nova-ST

NovaSeq flow cells are patterned with hexagonally packed oligonucleotide-functionalized nano-wells (Figure 1B). Compared to MiSeq flow cells, which are not patterned, the nano-functionalized surfaces of NovaSeq flow cells have several benefits, including higher spatial density and lower cluster crosstalk. We imaged the functionalized surface of a NovaSeq 6000 S4 flow cell with electron microscopy, revealing nano-wells of ~300 nm diameter and a well-to-well pitch of ~625 nm (Figure 1B), providing a resolution of ~350 nano-well spots for RNA capture per 100 μm^2 area. Similar to Seq-Scope,[10] Nova-ST starts with sequencing of high-density molecular indexing (HDMI) oligonucleotides, which contain Illumina sequencing adapters, a 32-base long randomer spatial identifier sequence, and a DraI restriction enzyme cleavable RNA capture site (Figure 1C; STAR Methods). The spatial coordinates on the flow cells are identified during the first sequencing pass, during which clonal clusters of HDMI molecules are generated by local cluster amplification of a single HDMI molecule in individual nano-wells (Figures 1C and S1A–S1D; details in the STAR Methods). This resulted in ~80% of the nano-wells receiving a unique HDMI spatial barcode. The resulting data from the HDMI sequencing were processed, whereby HDMI sequences and their intra-tile coordinates were extracted from demultiplexed FASTQ files and stored in binary files for downstream usage (further details in the STAR Methods). The preliminary HDMI sequencing quality, including the base composition, was assessed prior to downstream steps (Figure S1E). After HDMI sequencing, the flow cells were subjected to a series of enzymatic treatments to prepare them for spatial transcriptomics profiling. Firstly, to expose the RNA capture site of the HDMI clusters, overnight incubation with the DraI restriction enzyme was performed. Subsequently, treatment with a cocktail of exonuclease and phosphatase resulted in the removal of leftover sequencing adapters without an HDMI (Figure 1C).

Next, the flow cell was prepared for spatial transcriptomics capture (Figure 1D). The flow cell consists of three sandwiched layers: an upper thin glass layer, a gasket layer providing clearance for the fluidic flow and a lower thick glass layer (Figure 1D). The inner surfaces on both the thin and thick glass layers have functionalized nano-wells, and both surfaces are used during sequencing (Figure 1D). For cutting Nova-ST chips from the large NovaSeq S4 flow cells, we developed two different strategies. The first strategy is manual glass cutting, where we begin by separating the thin and thick glass layers. Once separated, the glass layers are scribed with a portable computer numerical control (CNC) equipped with a diamond tipped scribing tool (Figure 1D; details in the STAR Methods) along a rectangular grid of 1 × 1 cm (Figure 1D). This can also be performed using common glass scribing tools (as described elsewhere[13]) in place of using a CNC machine. After this, standard glass manipulation tools were used to break the large piece of glass along the scribed lines, separating it into individual Nova-ST spatial chips. A fully successful disassembly yields 36 thick and 36 thin 1 × 1 cm Nova-ST chips from a single NovaSeq S4 flow cell (Figure 1D). However, the manual cutting strategy is challenging; in particular, cutting of the thicker sections can result in uneven cuts and damage to the functional surface.

To overcome this challenge, we also developed a second, automated glass cutting strategy. In this method, the whole NovaSeq S4 flow cell is diced using a wafer dicing instrument (DISCO DAD 3220) into rectangular grid patterns of any desired dimensions (Figure 1E). The DAD 3220 employs an extremely thin diamond blade that physically saws the glass substrate while producing cut grooves in the range of just 200 μm. This dicing method provides a robust and very consistent workflow for producing Nova-ST chips compared to the manual cutting workflow. In the dicing method, the separation of the thin and thick glass layers is performed after dicing (Figure 1E), resulting in significantly reduced breakage and wastage. Due to the flexibility of dicing, any chip dimensions can be created. In this work, we prepared several different chip sizes (5 × 8 mm, 6 × 8 mm, and 10 × 8 mm) (Figure 1E). Following either cutting method, Nova-ST chips were prepared for RNA capture after a series of washes and chemical treatment steps (STAR Methods; Figures 1D, 1E, S1F, and S1G).

Implementation of spatial transcriptomics workflow

To test Nova-ST chips, we developed a spatial transcriptomics workflow for analyzing 10 μm fresh-frozen tissue sections. The cryosections were placed on the functional RNA capture area of the Nova-ST chips (Figure 2A). To perform tissue morphology registration for subsequent downstream alignment of the spatial data, hematoxylin and eosin (H&E) staining was performed on methanol-fixed tissue. The spatial RNA footprint of the tissue was created by enzymatic permeabilization, RNA capture, and first-strand synthesis during reverse transcription on the HDMI-functionalized surface of the Nova-ST chip (details in the STAR Methods; Figures 2A, S1H, and S1I). Subsequent second-strand synthesis and denaturation followed by PCR amplification yielded libraries ready for next-generation sequencing (STAR Methods; Figures 2A and S1J). Paired-end sequencing of these libraries resulted in information about the spatial coordinates via the HDMI barcode and the gene expression per individual barcode (details in the STAR Methods).

During initial HDMI sequencing on the NovaSeq instrument, the sequencer images the flow cell in small contiguous sections called tiles, and the coordinates of each sequenced cluster are reported within the context of these tiles rather than the whole flow cell. As these tiles are not physical features on the flow cell, it is not known *a priori* which Nova-ST chip contains which tiles. Therefore, in the Nova-ST analysis pipeline (Figure 2B), we begin with the identification of these tiles by processing a small portion of the reads from the spatial transcriptomics library and comparing them with a subset of the barcodes sequenced per tile during HDMI generation. Next, we extract all barcodes, as well as their spatial coordinates, from the identified tiles to create a barcode whitelist that is then used for gene expression quantification using STARsolo[14] (see the STAR Methods; Figure 2B).

The NovaSeq flow cells also contain a pattern of physical fiducial markings that are visible under a microscope as well as in the spatial coordinates due to their lack of nano-wells and, therefore,

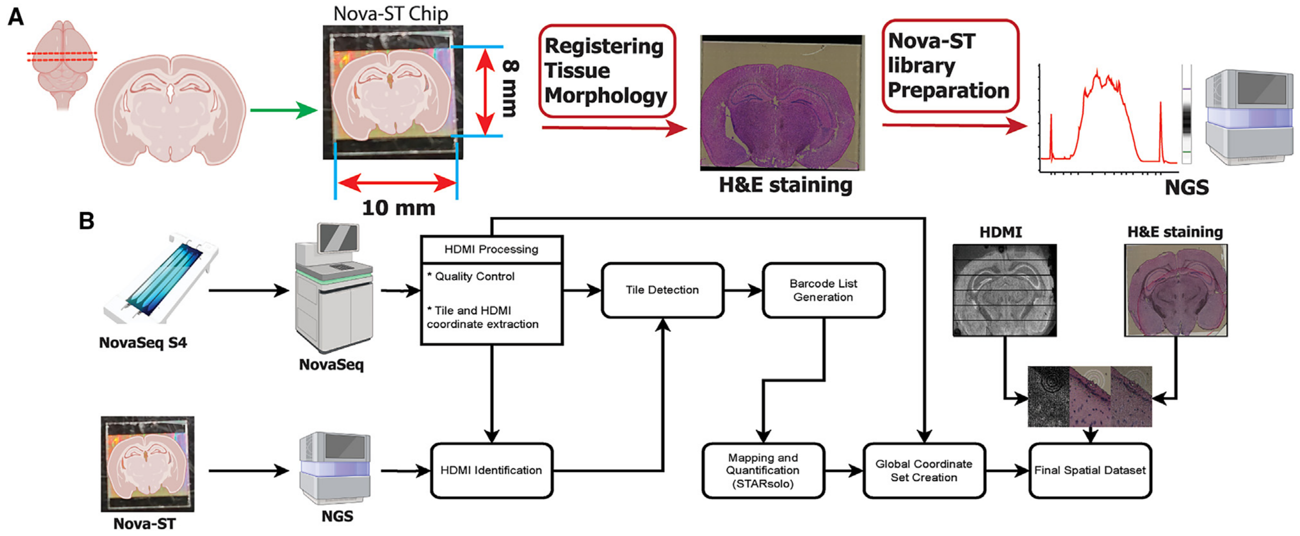

Figure 2. Overview of the spatial profiling with Nova-ST platform

(A) Experimental steps in spatial transcriptomics profiling of a tissue with Nova-ST. This tissue section on Nova-ST chip is stained with H&E for the subsequent downstream tissue registration. After the final library preparation, the quality of the library is analyzed and sequenced.

(B) A flow chart showing a schematic overview of the data generation and pre-processing steps in the Nova-ST pipeline.

HDMIs. Using the known distance between these fiducials (measured with light and electron microscopy), we updated the coordinates of HDMIs within each tile, placing them all within a global space of the Nova-ST chip. As this space is grounded in real measurements, we were able to align H&E images of the tissue section to the transcriptomic data using a simple affine transformation based solely on the fiducial markers (Figure 2B).

Performance assessment of Nova-ST

The final HDMI footprint of the tissue indicated high spatial resolution (Figures 3A and 3B), which is also shown by the inset showing a zoomed-in area. We performed seven replicates across two batches of Nova-ST chips created from two separate NovaSeq S4 flow cells. Replicates 1–5 are from one flow cell (FC1), and replicates 6 and 7 are from a second flow cell (FC2) (Figure 3C). The quality of the Nova-ST libraries is consistent between the chips derived from different flow cells as well as between the thick and thin glass sections of the flow cells (Figures 3A–3C). One random replicate was chosen for deep sequencing (~1.2 billion reads), while multiple shallowly sequenced replicates clearly indicate the robust and consistent performance of the workflow (Figures 3A–3C). The spatial resolution of the RNA footprint is similar between chip replicates from the same flow cell and between chips derived from different flow cells (Figures 3A and 3B). The performance, consistency, and reproducibility between different Nova-ST chips derived from both the inter- and intra-S4-sequencing flow cells are also evident from the quality metrics of the spatial data (Figure 3C).

The data were binned into contiguous chunks of 25 × 25 μm (bin50), 50 × 50 μm (bin100), and 100 × 100 μm (bin200) for pre-processing (see the STAR Methods). Figure 3D shows the spatial expression of several marker genes from both the Allen Brain Atlas (Merscope[15] and *in situ* hybridization [ISH] data[16]) and Nova-ST. Expression of each gene is localized in the correct brain region, such as *Lamp5* in the cortex and *Bcl11a* in the hippocampus, and spatial patterning of each gene is highly concordant between the Allen Brain Atlas and Nova-ST data. Further marker genes showing spatially localized expression are shown in Figure S2A.

Next, we assessed the reproducibility of Nova-ST by comparing quality metrics across the seven replicates (six at lower sequencing depth [77–140 million reads]) (Figure 3B). The percentage of reads with valid barcodes that were also successfully mapped ranged between 77.3% and 85.1%, and the percentage of unique molecular identifiers (UMIs) ranged between 36.48% and 78.60% (depending upon sequencing depth). For the three different bin sizes, bin50, bin100, and bin200, we detected medians of 994, 2,821, and 6,318 genes with non-zero counts and 2,131, 8,268, and 32,317 UMIs per bin for the deeply sequenced (DS) samples, respectively; for the shallowly sequenced samples, we detected medians of 263, 915, and 2,594 genes, with medians of 393, 1,613, and 6,233 UMIs, respectively (Figures 3E and S2B). Comparing total counts per gene across the filtered datasets at bin50 shows high reproducibility between samples (Figure S3; Table S1), and comparing summed gene counts between samples shows no batch effect between Nova-ST chips made from either flow cell (Figure S4A).

Unbiased clustering of bin50 data identified individual layers in the mouse cortex as well as subtypes within the hippocampus (Figure 3F). This resolution also allowed the identification of clusters of cells spanning just 1–2 cell layers (Figure S4B), such as layer 6b neurons, which formed a separate cluster, and agree with *Ccn2* (a layer 6b marker gene) expression shown in Figure S4C. To test whether the unbiased clustering corresponded to known cell types, we used cell2location[18] to map an independent single-cell RNA sequencing (scRNA-seq) dataset of the

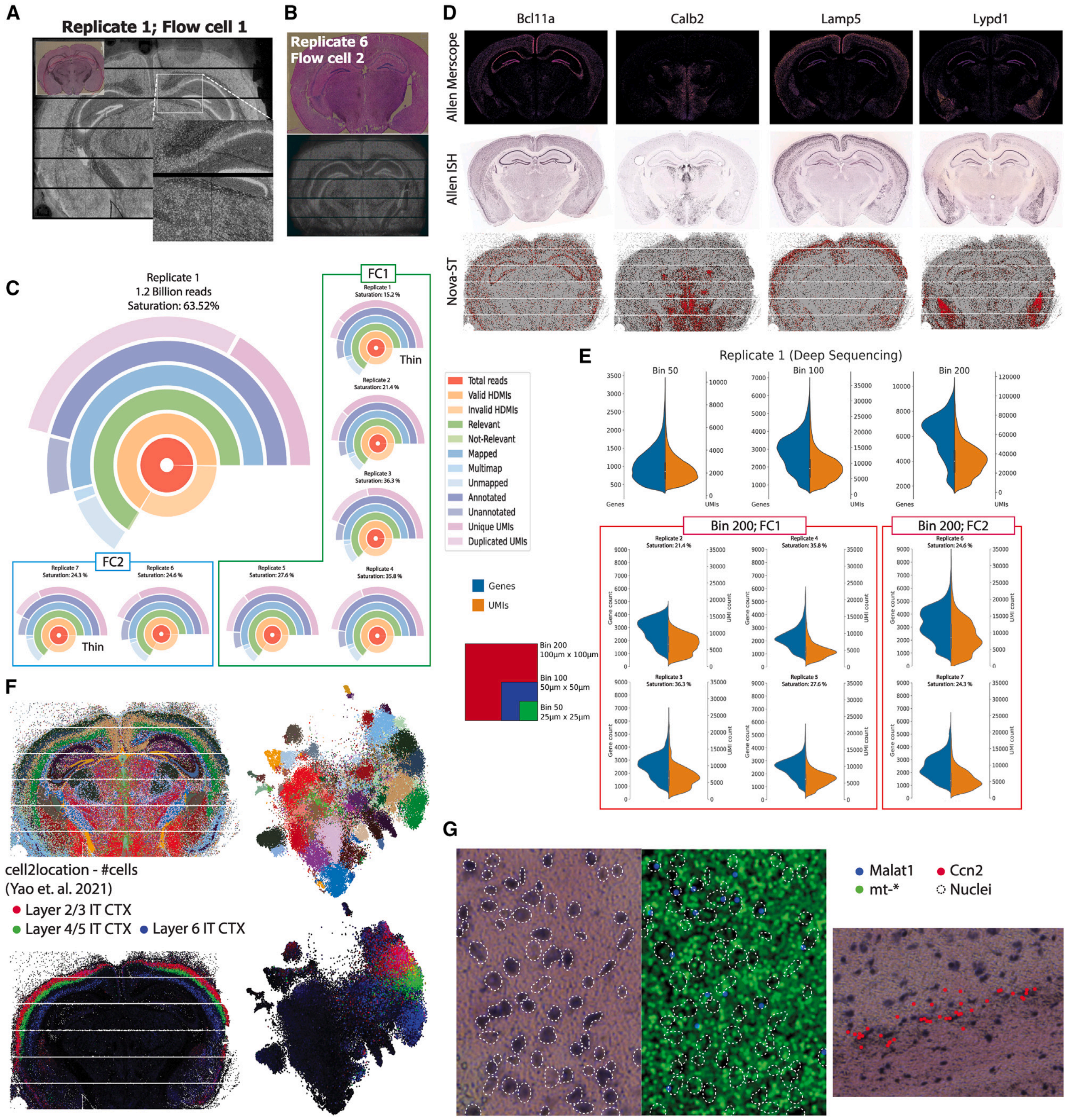

Figure 3. Performance of Nova-ST platform using mouse brain section

(A) Spatial HDMI footprint of the transcripts captured from the mouse brain section in the deeply sequenced (DS) sample—replicate 1 (FS1). H&E-stained tissue is present in the inset.

(B) Spatial HDMI footprint of the transcripts captured from the mouse brain and the H&E staining of the tissue of replicate 6 (FS2).

(C) Circle plots showing various metrics from the Nova-ST libraries for all mouse brain section replicates analyzed. Relevant and non-relevant have no counterpart in Nova-ST data but are included for consistency with Stereo-seq samples.

(D) Spatial gene expression mapping of several region-specific gene markers: Allen Brain Atlas data (top: Merscope[15] [Allen Brain Cell Atlas, https://knowledge.brain-map.org/abcatlas], middle: ISH[16] [Allen Mouse Brain Atlas, http://mouse.brain-map.org]) and Nova-ST (bottom visualized with SCope[17]).

(E) Violin plots of both number of genes detected and number of UMIs detected at bins 50, 100, and 200 for the DS replicate and bin 200 for all the remaining shallow sequenced replicates.

(legend continued on next page)

mouse cortex and hippocampus to the binned Nova-ST data.[19] This correctly identified the location of all annotated scRNA-seq cell types within the cortex and hippocampus, and cell types overall corresponded with the unbiased clustering (e.g., layer 2/3, layer 4/5, and layer 6 intra-telencephalic neurons shown in Figure 3F). Next, we assessed RNA diffusion during tissue permeabilization. For this, we localized mitochondrial mRNAs and nuclear RNA (*Malat1*) and found these transcripts to be largely non-overlapping, suggesting low levels of RNA diffusion (Figure 3G). This was also the case with cortical layer 6b in the cortex, where the expression of the *Ccn2* gene is localized to the 1- to 2-cell-layer-thick cortical region (Figure 3G).

Comparison of Nova-ST with existing sequencing-based ST technologies

Finally, we compared the performance (percentage of usable reads), sensitivity (UMI, gene counts, and capture efficiency), and the specificity (correct localization of scRNA-seq cell types by cell2location[18]) of Nova-ST with other sequencing-based spatial transcriptomics technologies.

First, we compared Nova-ST to other methods while controlling for sequencing depth. To this end, we performed a Stereo-seq experiment (Stereo-seq in-house [I.H.]) on a comparable mouse brain section and downsampled the Stereo-seq data to the same depth as the deeply sequenced Nova-ST run. Nova-ST libraries have similar metrics to Stereo-seq libraries with regards to mapping percentage and annotated reads (83.51% and 73.04% [Nova-ST] vs. 77.9% and 62.55% [Stereo-seq]) (Figures 3C and 4A). Nova-ST shows a lower percentage of recovered barcodes, a large portion of which are explained by sections of the chips that are not sequenced in the initial NovaSeq run but are still functional (visible as rows/columns with no counts in Figure 3A). Nova-ST has superior complexity, allowing more genes/UMIs to be detected at lower sequencing depths and greater information obtained in deeper sequencing (25.54% [Nova-ST] vs. 9.85% [Stereo-seq] unique UMIs at equal depth) (Figures 3C and 4A). At a bin size of 200, Nova-ST detects a median of 6,318 genes compared to 4,092 genes with the Stereo-seq platform at the same bin size (Figures 3E and 4B).

We also binned our Nova-ST data in 10 × 10 μm bins (bin20) to compare them with previously published Slide-seqV2 data[6,20] and another publicly available Stereo-seq sample[8] (Table 1; Figure 4C). We found that at a similar depth, we outperformed our I.H. Stereo-seq sample: we detect greater numbers of genes and UMIs than Slide-seqV2 (albeit with greater sequencing depth), and despite having over 4 times the number of reads and covering half the amount of tissue, when compared with the public Stereo-seq sample, we detect only ~20% fewer genes.

For Nova-ST, we detect a median UMI count of 294 per 100 μm^2 at a sequencing depth of ~15 million reads per mm^2 of total sequenced surface (deeply sequenced sample), which compares to the sensitivity reported by the Open-ST platform,[13] another recent spatial technique independently developed. Thus, Nova-ST obtains more genes and UMIs than Stereo-seq at similar bin sizes, allowing us to decrease the bin sizes in Nova-ST to achieve increased resolution (Figures 3E and 4B).

Next, we compared the per-gene capture efficiency of Nova-ST to other methods without correcting for sequencing depth for a head-to-head comparison. We selected a similar brain area of Nova-ST, Visium HD (10× Genomics), and three Stereo-seq (BGI STomics) datasets: the I.H.-produced dataset, the Stereo-seq dataset from Chen et al.,[8] and the Stereo-seq dataset from You et al.[20] (Figure 4D). The selected area consisted of the cortex, corpus callosum, and cornu ammonis 1 of the hippocampus. The Nova-ST gene counts correlated well with other technologies, with Spearman's correlations of 0.83, 0.93, 0.92, and 0.95 for Visium HD (Figure 4H), Stereo-seq I.H. (Figure 4I), Stereo-seq Chen et al. (Figure 4J), and Stereo-seq You et al. (Figure 4K), respectively. The lower correlation of Visium HD is due to it being a targeted, probe-based method that does not include all genes (Figure 4F). Comparing the detected genes, Visium HD has slightly higher counts with a median fold improvement of 1.57 (Figure 4L). Compared to Stereo-seq, Nova-ST falls in the range of detection efficiencies, where it measures a median of 2.05 times higher counts than the I.H. dataset and 1.11 times higher than the Chen et al. dataset, even though these were sequenced to equal or greater depth. Nevertheless, the You et al. dataset captures more transcripts per area, with a median gene count 2.62 times higher, albeit with 2.6 times more reads (Figure 4L). Thus, performance is dependent on sequencing depth, yet with less reads, Nova-ST still obtains higher counts.

To estimate the amount of RNA captured by Nova-ST and other sequencing-based methods, we compared counts in the selected area to high-sensitivity osmFISH data[21] (Figure 4E). We observe that all sequencing-based methods capture just a few percent of the total RNA that is present in the tissue sample. Without normalizing the sequencing depth of the different spatial datasets (Figure 4G), we observe median gene detection efficiencies of 1.08% for Nova-ST, 1.3% for Visium HD, and 0.43%, 0.79%, and 2.48% for the Stereo-seq datasets (Figure 4F). Finally, unbiased clustering of our I.H. Stereo-seq sample at bin50 shows some spatial localization of clusters, although without the ability to clearly delineate cortical layers or subtypes in the hippocampus (Figure 4M). When performing the cell2location analysis as described above, cortical layers are visible; however, they are both diffuse and contain high levels of background signals across the tissue section (Figure 4N) when compared to the predictions on our Nova-ST data (Figure 3F).

Future perspectives of Nova-ST using NovaSeq X chips

Illumina recently introduced the next generation of NovaSeq sequencers, namely the NovaSeq X and X Plus systems, which may eventually replace the NovaSeq 6000 series. Illumina offers three different X-series flow cells: (1) 1.5 billion reads (X-1.5B), (2) 10 billion reads (X-10B), and (3) 25 billion reads (X-25B). Compared to the other flow cells, the X-25B flow cells are most suitable for

(F) t-Distributed stochastic neighbor embedding (t-SNE) and spatial visualizations for Nova-ST DS showing Leiden clustering. Cell2location[18] predictions for different layers in the cortex are also displayed.

(G) Two areas of tissues showing (1) Malat1 expression localized to cell nuclei, distinct from mitochondrial reads, and (2) Ccn2 expression limited to cortical layer 6b.

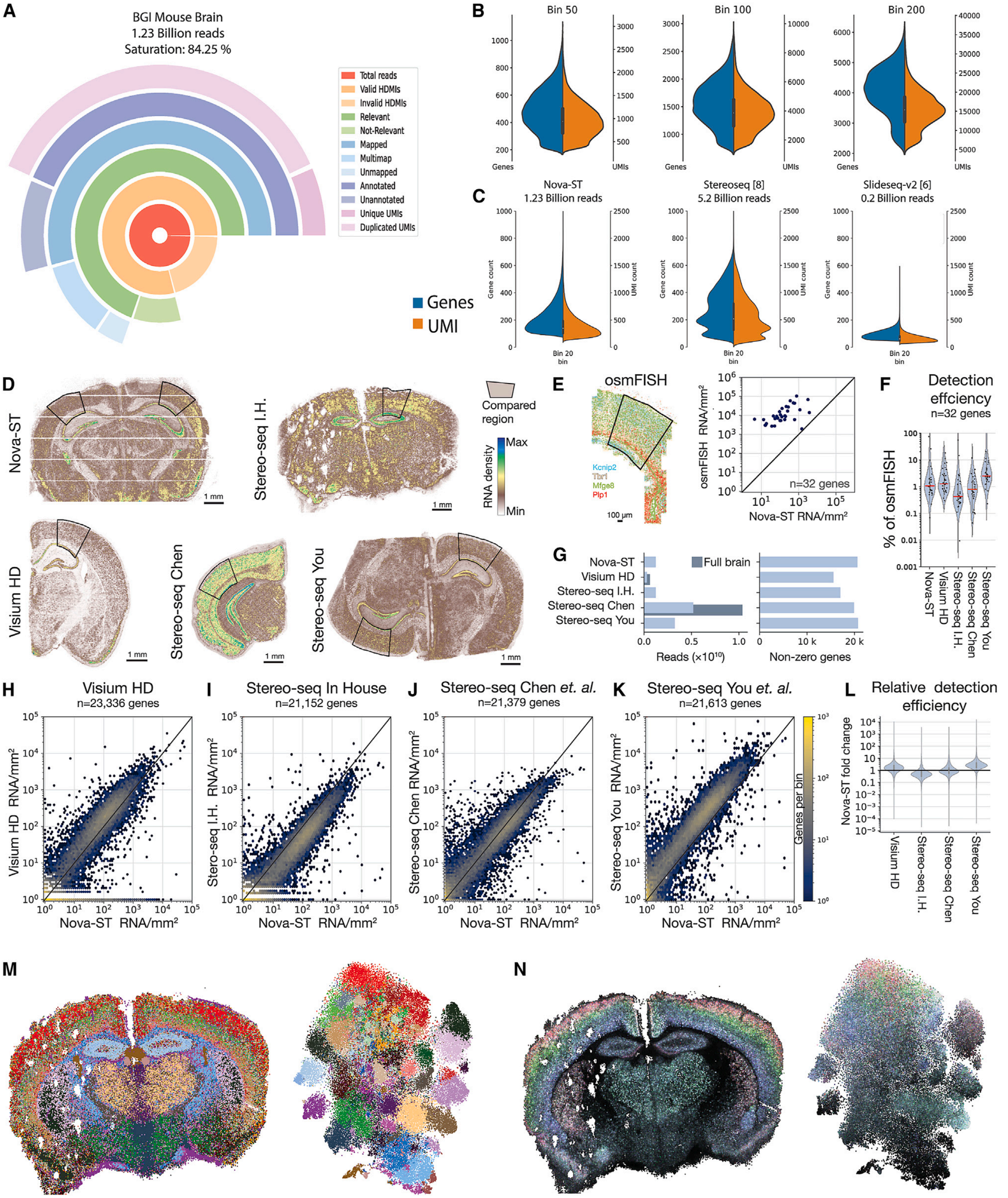

Figure 4. Comparison of Nova-ST with other spatial transcriptomics technologies

(A) Circle plot displaying the sequencing metrics of a Stereo-seq coronal mouse brain section downsampled to 1.23 billion reads to match the Nova-ST DS sample.

(legend continued on next page)

Nova-ST due to the wider channel dimension (~6 mm, Figure S5A) supporting profiling of large tissue sections. Since the automatic dicing strategy worked best for cutting and separating NovaSeq 6000 flow cells, we used this to dice a (used) X-25B flow cell, yielding ~56 diced chips (Figure S5B). As with the NovaSeq 6000 flow cells, each section of the diced flow cell can subsequently be separated into 2 separate chips, where the inner surfaces are functional. Successful dicing and layer separation yielded a total of ~112 chips with an approximate dimension of 6 × 9 mm for spatial RNA profiling (Figure S5B).

Characterization with electron microscopy revealed the functional surface of the NovaSeq X-25B chip to have a denser pattern of nano-wells compared to the NovaSeq 6000 flow cells. Analysis indicated the nano-well size to be ~280 nm with a pitch between nano-wells of ~399 nm (Figure S5C), translating into a ~3.5× fold reduction in the dead space for the NovaSeq X-25B flow cells compared to the S4 flow cells (Figure S5D). This increased density of nano-wells will likely enhance the transcript capture and, in turn, further improve the capture sensitivity and spatial resolution of Nova-ST. In conclusion, we demonstrate that Nova-ST can also be adapted to work with the new generation of NovaSeq X flow cells.

DISCUSSION

Spatial analysis is becoming a commonplace method in many fields of biomedical research, including cancer biology, neuroscience, and developmental biology.[4] Sequencing-based spatial barcoding workflows offer flexibility, ease of use, and the ability to perform unbiased spatial transcriptomics on any desired tissue and species. Existing sequencing-based technologies have several limitations, such as spatial resolution, area for spatial capture, and financial feasibility of profiling large-scale tissue sections. To overcome some of these challenges, we have developed Nova-ST, a workflow that uses spatially barcoded nano-patterned Illumina sequencing flow cells to perform spatial transcriptomics.

To enable large-throughput spatial screening, we chose the NovaSeq S4 flow cell for developing Nova-ST. To prepare Nova-ST chips from randomly barcoded NovaSeq flow cells, we devised two different cutting strategies, namely (1) manual manipulation and (2) an automated glass dicing technique. We found both methods to be robust, producing many Nova-ST chips from a single flow cell, yet the automated method proved more reliable, allowing for further size customizations as well as minimal breakage and wastage. Furthermore, the automated dicing strategy also resulted in minimal damage or artifacts to the functional surface of the chips.

To demonstrate the performance of Nova-ST chips and benchmark it against other spatial barcoding technologies, we developed a spatial transcriptomics workflow and used commonly used coronal mouse brain cryosections. Nova-ST uses standard H&E staining for spatial registration of the tissue and the subsequent downstream alignment of the spatial data. We developed a computational workflow that rapidly processes both the initial NovaSeq data and the spatial libraries to produce easy-to-use files for downstream processing and registration while also providing multiple quality control (QC) steps for assessing data and spatial library quality.

Spatial analysis on mouse brain sections indicated the superior performance of Nova-ST. Quality metrics show Nova-ST libraries to have a wide transcript diversity and display consistency and reproducibility between chips derived from the same NovaSeq S4 flow cell as well between chips derived from different flow cells. Notably, binned gene expression patterns of several gene markers were localized in the expected brain regions with near single-cell resolution. Expression of the gene Ccn2, for example, is localized to cells of layer 6b, just 1–2 cell layers thick of cortex; this is recapitulated across multiple independent replicates. Unbiased clustering of binned Nova-ST data robustly identified individual layers of the cortex and other brain regions, further confirmed by cell2location[18] predictions using an independent scRNA mouse cortex dataset.

To compare and benchmark the performance of Nova-ST to other spatial technologies, we compared Nova-ST with mouse cortex Stereo-seq data from both the I.H. sample and published data[8,20] and hippocampus data from Slide-seqV2[6] and Visium HD (publicly available data). When compared to the I.H.-generated Stereo-seq data, Nova-ST displays superiority in performance with higher gene detection sensitivity and a higher gene/UMI ratio detected at lower sequencing depths. When compared to other publicly available spatial datasets derived from various spatial sequencing technologies, Nova-ST displays higher or comparable performance. Finally, we show that the recently released NovaSeq X flow cells are compatible with Nova-ST, possibly providing a greater spatial resolution as well as higher sensitivity due to the increased density of the functionalized area.

Open-ST[13] is another, independently developed, spatial transcriptomics technique based on the NovaSeq 6000 S4 flow cell and is very comparable to Nova-ST. Nevertheless, there are some key differences between the two implementations. Firstly, on the experimental side, the automatic dicing in Nova-ST allows for the robust production of larger chips with minimal artifacts. Secondly, on the computational side, the Nova-ST pipeline aligns the spatial data and H&E staining in a single step, whereas in Open-ST, each tile is aligned with the H&E image individually.

(B) Violin plots of bin50, bin100, and bin200 datasets showing the distribution of the number of genes and UMIs detected across bins for the in-house BGI Stereo-seq sample.
(C) Violin plots of gene and UMI counts across bin20 (10 × 10 μm) data from Nova-ST (this study), Stereo-seq,[8] and Slide-seqV2.[6]
(D) Sequencing-based ST datasets with selected region for comparison.
(E) osmFISH dataset with compared region; transcripts per area are compared to Nova-ST.
(F) Distribution of gene detection efficiencies comparing osmFISH to the sequencing-based methods.
(G) Summary plot of number of reads and detected genes per dataset. Dark blue bar indicates projected read depth corrected for a full mouse brain section.
(H–L) Per-gene detection efficiency for all sequencing-based methods compared to Nova-ST.
(M) Spatial and t-SNE visualizations of Leiden clustering (resolution 4.0) on our in-house Stereo-seq data.
(N) Spatial and t-SNE visualizations of cell2location-predicted number of cells for cortical layer neurons (legend in Figure 3F).

Table 1. Sequencing metrics from various sequencing-based spatial transcriptomics technologies at bin20 (10 × 10 μm)

Sample	Area covered	Reads (billion)	Median genes	Median UMIs
Nova-ST	entire coronal section	1.23	199	349
In-house Stereo-seq	entire coronal section	1.23	91	155
Slide-seqV2[6,19]	hippocampus only	~0.2	153	211
Stereo-seq[8]	half coronal section	5.2	266	519

Our approach reduces the risk of producing alignments that are not consistent with real-world distances. In addition, Nova-ST generates a whitelist of barcodes for each tile based on the original HDMI sequencing, which is then matched with barcodes discovered in the RNA sequencing (allowing for 1 mismatch). In Open-ST, all barcodes present within the RNA data are counted without comparison to a whitelist; this may result in decreased sensitivity when errors are introduced during sequencing. Despite these differences, Open-ST confirms the QC and benchmarking statistics we observed with Nova-ST, suggesting that spatial transcriptomics with Illumina chips provides robust, cost-effective, and sensitive data.

Unlike the recently introduced 10× Genomics Visium HD, Nova-ST is species agnostic and can also be used for studying non-model organisms. Nova-ST costs (including the reagents for first-strand synthesis and library prep) approximately 7.5€ per mm^2 of spatial area, excluding the cost of sequencing. This is approximately 17% of the list prices (~45€ per mm^2) of both the BGI Stereo-seq and 10× Genomics Visium HD platforms. This shows that Nova-ST not only outperforms the nearest competitive technology in terms of its performance in spatial profiling but will also pave way for cost-effective profiling of large-scale spatial transcriptomics. Due to the open-source flexibility of Nova-ST, further development for integrating other multiomics modalities such as asssay for transposase-accessible chromatin using sequencing (ATAC-seq) or spatial protein expression becomes a possibility, thus allowing for the broader use of Nova-ST and adoption within the scientific community for larger-scale tissue genomics.

Limitations

A potential limitation related to Nova-ST is that it requires access to an Illumina NovaSeq sequencer. For labs with no direct access to such a sequencer, it is possible for a sequencing core facility to generate an HDMI barcoded flow cell and transport the chip as a cold pack shipment. A second limitation is that chip cutting requires certain pieces of equipment, either a CNC equipped with a diamond-tipped scribing tool or a wafer dicing instrument for Nova-ST or a 3D-printed guide and diamond-tipped scribing tool for Open-ST. While manual cutting is the most accessible, it requires some training on used Illumina chips to prevent uneven cuts or loss of chips; the thicker glass side is particularly prone to uneven breaking. Downstream handling with unevenly cut chips can be challenging and may prohibit certain tissue sizes and/or shapes from being profiled. We recommend automatic dicing when specialized instrumentation is available. A third limitation is that, in the current form, Nova-ST is not suitable for full-length transcriptome analysis. We envision that with the slight modification of introducing a template switching oligonucleotide-based first-strand synthesis,[22,23] full-length transcripts could be obtained, which, in combination with long-read sequencing platforms such as Oxford Nanopore or PacBio, could be used to interrogate splicing and isoform dynamics in tissue with a spatial context. A final limitation of Nova-ST is that the data analysis as presented here is based on a binning strategy; when combining contiguous regions of clusters together, this aggregation can lead to loss of single-cell resolution. Nuclei-based cell segmentation could be used to alleviate this problem, likely with a small decrease in sensitivity. A more elegant solution currently being investigated is the use of cell boundary staining to demarcate the cell boundaries. Despite certain limitations, Nova-ST is an easy-to-implement and, most importantly, cost-effective way of performing sequencing-based spatial transcriptomics at a higher or equivalent sensitivity compared to competing commercial methods.

STAR★METHODS

Detailed methods are provided in the online version of this paper and include the following:

- KEY RESOURCES TABLE
- RESOURCE AVAILABILITY
  - Lead contact
  - Materials availability
  - Data and code availability
- EXPERIMENTAL MODEL AND STUDY PARTICIPANT DETAILS
- METHOD DETAILS
  - Mouse tissue preparation for cryo-sectioning
  - Stereo-seq optimization of tissue permeabilization
  - Stereo-seq spatial transcriptomics analysis
  - HDMI sequencing
  - Post sequencing processing of the flow cell
  - Flow cell disassembly for Nova-ST chip preparation
- QUANTIFICATION AND STATISTICAL ANALYSIS

SUPPLEMENTAL INFORMATION

Supplemental information can be found online at https://doi.org/10.1016/j.crmeth.2024.100831.

ACKNOWLEDGMENTS

We thank Dr. Pieter Baatsen, Katlijn Vints, and Dr. Malgorzata Sliwinska from the electron microscopy platform at VIB KU Leuven for their help with scanning electron microscopy. We would also like to thank Donald Raddoux from Elektronische Circuits en Systemen, KU Leuven, for his invaluable support with the DISCO DAD 3220 wafer dicing platform. Parts of Figure 2 and the graphical abstract were created with BioRender.com. This work is partially funded by the following grants to S.A.: an ERC Advanced Grant (101054387_Genome2Cells), FWO grants (G044124N and G094121N), a KU Leuven grant (C14/22/125), and Aligning Science Across Parkinson's (ASAP-000430) through the Michael J. Fox Foundation for Parkinson's Research. L.E.B. is supported by an EMBO postdoctoral fellowship (ALTF 638-2023).

AUTHOR CONTRIBUTIONS

S.P., K.D., and S.A. conceived and developed the Nova-ST workflow; S.P. and K.D. performed the experiments and data analysis with help from R.V., L.E.B., N.P., A.V., and N.C.; S.P., K.D., and S.A. wrote the paper with input from all the other authors; and S.P. and S.A. provided supervision and guidance for the project.

DECLARATION OF INTERESTS

The authors declare no competing interests.

Received: April 9, 2024
Revised: June 7, 2024
Accepted: July 11, 2024
Published: August 6, 2024

REFERENCES

1. Vickovic, S., Eraslan, G., Salmén, F., Klughammer, J., Stenbeck, L., Schapiro, D., Äijö, T., Bonneau, R., Bergenstråhle, L., Navarro, J.F., et al. (2019). High-definition spatial transcriptomics for in situ tissue profiling. Nat. Methods *16*, 987–990. https://doi.org/10.1038/s41592-019-0548-y.
2. Lein, E., Borm, L.E., and Linnarsson, S. (2017). The promise of spatial transcriptomics for neuroscience in the era of molecular cell typing. Science *358*, 64–69. https://doi.org/10.1126/SCIENCE.AAN6827/ASSET/1FE1FBA8-1E6D-44F7-AF5A-D459C148EB0C/ASSETS/GRAPHIC/358_64_F3.JPEG.
3. Rao, A., Barkley, D., França, G.S., and Yanai, I. (2021). Exploring tissue architecture using spatial transcriptomics. Nature *596*, 211–220. https://doi.org/10.1038/s41586-021-03634-9.
4. Williams, C.G., Lee, H.J., Asatsuma, T., Vento-Tormo, R., and Haque, A. (2022). An introduction to spatial transcriptomics for biomedical research. Genome Med. *14*, 68. https://doi.org/10.1186/S13073-022-01075-1/FIGURES/3.
5. Salmén, F., Ståhl, P.L., Mollbrink, A., Navarro, J.F., Vickovic, S., Frisén, J., and Lundeberg, J. (2018). Barcoded solid-phase RNA capture for Spatial Transcriptomics profiling in mammalian tissue sections. Nat. Protoc. *13*, 2501–2534. https://doi.org/10.1038/s41596-018-0045-2.
6. Stickels, R.R., Murray, E., Kumar, P., Li, J., Marshall, J.L., Di Bella, D.J., Arlotta, P., Macosko, E.Z., and Chen, F. (2020). Highly sensitive spatial transcriptomics at near-cellular resolution with Slide-seqV2. Nat. Biotechnol. *39*, 313–319. https://doi.org/10.1038/s41587-020-0739-1.
7. Liu, Y., Yang, M., Deng, Y., Su, G., Enninful, A., Guo, C.C., Tebaldi, T., Zhang, D., Kim, D., Bai, Z., et al. (2020). High-Spatial-Resolution Multi-Omics Sequencing via Deterministic Barcoding in Tissue. Cell *183*, 1665–1681.e18. https://doi.org/10.1016/J.CELL.2020.10.026.
8. Chen, A., Liao, S., Cheng, M., Ma, K., Wu, L., Lai, Y., Qiu, X., Yang, J., Xu, J., Hao, S., et al. (2022). Spatiotemporal transcriptomic atlas of mouse organogenesis using DNA nanoball-patterned arrays. Cell *185*, 1777–1792.e21. https://doi.org/10.1016/J.CELL.2022.04.003.
9. Fu, X., Sun, L., Dong, R., Chen, J.Y., Silakit, R., Condon, L.F., Lin, Y., Lin, S., Palmiter, R.D., and Gu, L. (2022). Polony gels enable amplifiable DNA stamping and spatial transcriptomics of chronic pain. Cell *185*, 4621–4633.e17. https://doi.org/10.1016/J.CELL.2022.10.021.
10. Cho, C.S., Xi, J., Si, Y., Park, S.R., Hsu, J.E., Kim, M., Jun, G., Kang, H.M., and Lee, J.H. (2021). Microscopic examination of spatial transcriptome using Seq-Scope. Cell *184*, 3559–3572.e22. https://doi.org/10.1016/j.cell.2021.05.010.
11. Poovathingal, S., Davie, K., and Aerts, S. (2024). Nova-ST Spatial Transcriptomics protocol. Protocols.io. https://doi.org/10.17504/protocols.io.n92ld835ov5b/v1.
12. Poovathingal, S., Davie, K., and Aerts, S. (2024). Nova-ST Chip Preparation Protocol. Protocols.io.. https://doi.org/10.17504/protocols.io.3byl4925jgo5/v1.
13. Schott, M., León-Periñán, D., Splendiani, E., Strenger, L., Licha, J.R., Pentimalli, T.M., Schallenberg, S., Alles, J., Tagliaferro, S.S., Boltengagen, A., et al. (2023). Open-ST: High-resolution spatial transcriptomics in 3D. Preprint at bioRxiv. https://doi.org/10.1101/2023.12.22.572554.
14. Kaminow, B., Yunusov, D., and Dobin, A. (2021). STARsolo: accurate, fast and versatile mapping/quantification of single-cell and single-nucleus RNA-seq data. Preprint at bioRxiv. https://doi.org/10.1101/2021.05.05.442755.
15. Zhang, M., Pan, X., Jung, W., Halpern, A.R., Eichhorn, S.W., Lei, Z., Cohen, L., Smith, K.A., Tasic, B., Yao, Z., et al. (2023). Molecularly defined and spatially resolved cell atlas of the whole mouse brain. Nature *624*, 343–354. https://doi.org/10.1038/s41586-023-06808-9.
16. Lein, E.S., Hawrylycz, M.J., Ao, N., Ayres, M., Bensinger, A., Bernard, A., Boe, A.F., Boguski, M.S., Brockway, K.S., Byrnes, E.J., et al. (2007). Genome-wide atlas of gene expression in the adult mouse brain. Nature *445*, 168–176. https://doi.org/10.1038/nature05453.
17. Davie, K., Janssens, J., Koldere, D., De Waegeneer, M., Pech, U., Kreft, Ł., Aibar, S., Makhzami, S., Christiaens, V., Bravo González-Blas, C., et al. (2018). A Single-Cell Transcriptome Atlas of the Aging Drosophila Brain. Cell *174*, 982–998.e20. https://doi.org/10.1016/J.CELL.2018.05.057/ATTACHMENT/0C7CFF5D-CE56-4299-AA21-A84B0E039C55/MMC6.PDF.
18. Kleshchevnikov, V., Shmatko, A., Dann, E., Aivazidis, A., King, H.W., Li, T., Elmentaite, R., Lomakin, A., Kedlian, V., Gayoso, A., et al. (2022). Cell2location maps fine-grained cell types in spatial transcriptomics. Nat. Biotechnol. *40*, 661–671. https://doi.org/10.1038/s41587-021-01139-4.
19. Yao, Z., van Velthoven, C.T.J., Nguyen, T.N., Goldy, J., Sedeno-Cortes, A.E., Baftizadeh, F., Bertagnolli, D., Casper, T., Chiang, M., Crichton, K., et al. (2021). A taxonomy of transcriptomic cell types across the isocortex and hippocampal formation. Cell *184*, 3222–3241.e26. https://doi.org/10.1016/J.CELL.2021.04.021.
20. You, Y., Fu, Y., Li, L., Zhang, Z., Jia, S., Lu, S., Ren, W., Liu, Y., Xu, Y., Liu, X., et al. (2023). Systematic comparison of sequencing-based spatial transcriptomic methods. Preprint at bioRxiv. https://doi.org/10.1101/2023.12.03.569744.
21. Codeluppi, S., Borm, L.E., Zeisel, A., La Manno, G., van Lunteren, J.A., Svensson, C.I., and Linnarsson, S. (2018). Spatial organization of the somatosensory cortex revealed by osmFISH. Nat. Methods *15*, 932–935. https://doi.org/10.1038/s41592-018-0175-z.
22. De Rop, F.V., Ismail, J.N., González-Blas, C.B., Hulselmans, G.J., Flerin, C.C., Janssens, J., Theunis, K., Christiaens, V.M., Wouters, J., Marcassa, G., et al. (2022). HyDrop enables droplet based single-cell ATAC-seq and single-cell RNA-seq using dissolvable hydrogel beads. Elife *11*, e73971. https://doi.org/10.7554/ELIFE.73971.
23. Picelli, S., Faridani, O.R., Björklund, Å.K., Winberg, G., Sagasser, S., and Sandberg, R. (2014). Full-length RNA-seq from single cells using Smart-seq2. Nat. Protoc. *9*, 171–181. https://doi.org/10.1038/nprot.2014.006.
24. Dobin, A., Davis, C.A., Schlesinger, F., Drenkow, J., Zaleski, C., Jha, S., Batut, P., Chaisson, M., and Gingeras, T.R. (2013). STAR: ultrafast universal RNA-seq aligner. Bioinformatics *29*, 15–21. https://doi.org/10.1093/bioinformatics/bts635.
25. Wolf, F.A., Angerer, P., and Theis, F.J. (2018). SCANPY: large-scale single-cell gene expression data analysis. Genome Biol. *19*, 15. https://doi.org/10.1186/s13059-017-1382-0.
26. Schindelin, J., Arganda-Carreras, I., Frise, E., Kaynig, V., Longair, M., Pietzsch, T., Preibisch, S., Rueden, C., Saalfeld, S., Schmid, B., et al. (2012). Fiji: an open-source platform for biological-image analysis. Nat. Methods *9*, 676–682. https://doi.org/10.1038/nmeth.2019.

STAR★METHODS

KEY RESOURCES TABLE

REAGENT or RESOURCE	SOURCE	IDENTIFIER
Chemicals, peptides, and recombinant proteins		
UltraPure^{TM} DNase/RNase-Free Distilled Water	Thermo Fisher Scientific	Catalog #10977023
KAPA Library Quantification Kit for Illumina® Platforms	Kapa Biosystems	Catalog #KK4835
Sodium hydroxide, 10N aq. soln	Thermo Fisher Scientific	Catalog #J63736.AE
Tris-HCl, 1M Solution, pH 8.0, Molecular Biology Grade, Ultrapure	Thermo Fisher Scientific	Catalog #J22638.AE
DOW SYLGARD 184, 1.1KG Silicone Elastomer, Flowable, Sylgard® 184, RT Cure, Transparent, Container	Dow Corning	Catalog #101697
DraI - 10,000 units	New England Biolabs	Catalog #R0129L
TE pH 7.5	IDT Technologies	Catalog #11-01-02-02
Exonuclease I (E.coli) - 15,000 units	New England Biolabs	Catalog #M0293L
Quick CIP – 5,000 units	New England Biolabs	Catalog #M0525L
3M 201E 48MM Masking Tape, Crepe Paper, Cream, 48 mm × 50 m	3M corporation	Catalog #201E 48MM
RNaseZap®	Thermo Fisher Scientific	Catalog #AM9780
DNAZap PCR DNA Degradation Solutions	Thermo Fisher Scientific	Catalog #AM9890
Tris (1 M), pH 7.0, RNase-free	Thermo Fisher Scientific	Catalog #AM9851
Pepsin from porcine gastric mucosa	Merck MilliporeSigma (Sigma-Aldrich)	Catalog #P7000
Hydrochloric Acid Solution, 0.1N (N/10) (Certified)	Thermo Fisher Scientific	Catalog #7647-01-0
1x Maxima H- RT buffer	Thermo Fisher Scientific	Catalog #EP0753
Maxima H-RT enzyme	Thermo Fisher Scientific	Catalog #EP0753
dNTP Mix (25 mM each)	Thermo Fisher Scientific	Catalog #R1121
Methanol (100%)	Merck Millipore Sigma (Sigma-Aldrich)	Catalog #34860-1L-R
2-Propanol	Merck Millipore Sigma (Sigma-Aldrich)	Catalog #59304-100ML-F
RNase Inhibitor	Lucigen	Catalog #30281-1
Ficoll PM-400 20% in H2O	Merck MilliporeSigma (Sigma-Aldrich)	Catalog #F5415-50ML
UltraPure SSC, 20X	Thermo Fisher Scientific	Catalog #15557044
Klenow Fragment (3′-5′ exo-) - 1,000 units	New England Biolabs	Catalog #M0212L
NaCl (5 M), RNase-free	Thermo Fisher Scientific	Catalog #AM9760G
SDS, 10% Solution	Life Technologies	Catalog #AM9822
EDTA (0.5 M), pH 8.0	Life Technologies	Catalog #AM9260G
Proteinase K, Molecular Biology Grade - 2 mL	New England Biolabs	Catalog #P8107S
Ampure XP Beads	Beckman Coulter	Catalog #A63882
HotStart ReadyMix (KAPA HiFi PCR kit)	Kapa Biosystems	Kapa Biosystems
Buffer EB	Qiagen	Catalog #19086
Sodium Hydroxide Solution (10N/Certified)	Fisher Scientific Chemicals	Catalog # SS255-1
1X TE buffer	IDT Technologies	Catalog # 11-05-01-09
SSC Buffer, 20X Concentrate	Merck Millipore Sigma (Sigma-Aldrich)	SRE0068-1L
Mayer’s haematoxylin	Agilent	S3309
Bluing buffer	Agilent	CS702
Eosin-Y	Merck Millipore Sigma (Sigma-Aldrich)	HT110216
Critical commercial assays		
Illumina NovaSeq 6000 S4 kit; 35 cycles	Illumina, Inc	Catalog #20044417
Visium Spatial Gene Expression Reagent Kits	10x Genomics, Inc	Catalog #1000192

(*Continued on next page*)

Continued

REAGENT or RESOURCE	SOURCE	IDENTIFIER
Stereo-seq Permeabilization kit	BGI STomics	Catalog #111KP118
Stereo-seq Chip P (1cm*1cm)	BGI STomics	Catalog #111CP118
Stereo-seq Transcriptomics T kit	BGI STomics	Catalog #111KT114
Stereo-seq Chip T (1cm*1cm)	BGI STomics	Catalog #110CT114
Stereo-seq Library Prep kit	BGI STomics	Catalog #111KL114
2100 Bioanalyzer – High sensitivity DNA analysis	Agilent Technologies	Catalog #G2939BA
Qubit^{TM} dsDNA Quantification Assay Kits	Thermo Fisher Scientific	Catalog #Q32851
Qubit^{TM} ssDNA Assay Kit	Thermo Fisher Scientific	Catalog #Q10212
innuPREP mini RNA kit	Analytik Jena	Catalog # AJ 845-KS-2040250
Deposited data		
Nova-ST Data (Mouse Brain)	This study	GEO: GSE256318
Stereo-seq (Mouse Brain)	This study	GEO: GSE256319
Stereo-seq (Mouse Brain)	Chen et al. (2022)[8]	CNGB: CNS0390078
Stereo-seq (Mouse Brain)	You et al. (2023)[20]	https://www.genographix.com
Slideseq-v2 (Mouse Hippocampus)	Stickels et al. (2021)[6]	https://singlecell.broadinstitute.org/single_cell/study/SCP815
10X Genomics and SMART-seq2 (Mouse Brain)	Yao et al. (Cell)[19]	https://portal.brain-map.org/atlases-and-data/rnaseq/mouse-whole-cortex-and-hippocampus-10x
Experimental models: Organisms/strains		
Six to eight weeks old mice (C57BL/6J)	JAX	MGI:3028467
Oligonucleotides		
HDMI-DraI32	IDT Technologies (Standard desalted)	CAAGCAGAAGACGGCATACGAGATTCTTTCCCTACACGACGCTCTTCCGATCTNNVNNVNNVNNVNNVNNNNNTCTTGTGACTACAGCACCCTCGACTCTCGCTTTTTTTTTTTTTTTTTTTTTTTTTTTTTTAAAGACTTTCACCAGTCCATGATGTGTAGATCTCGGTGGTCGCCGTATCATT
Read1-DraI32	IDT Technologies (PAGE purification)	ATCATGGACTGGTGAAAGTCTTTAAAAAAAAAAAAAAAAAAAAAAAAAAAAAAGCGAGAGTCGAGGGTGCTGTAGTCACAAGA
RPE_Randomer	IDT Technologies (Standard desalted)	TCAGACGTGTGCTCTTCCGATCTNNNGGNNNB
RPEPCR_Fwd	IDT Technologies (Standard desalted)	TCTTTCCCTACACGACGC*T*C
RPEPCR_Rev	IDT Technologies (Standard desalted)	TCAGACGTGTGCTCTTCC*G*A
P5_WTA_TCAAGCGA	IDT Technologies (Standard desalted)	AATGATACGGCGACCACCGAGATCTACACTCAAGCGAACACTCTTTCCCTACACGACGCTCTTCCGATCT
P5_WTA_ GTAGCTGA	IDT Technologies (Standard desalted)	AATGATACGGCGACCACCGAGATCTACACGTAGCTGAACACTCTTTCCCTACACGACGCTCTTCCGATCT
P5_WTA_ CCAACCAA	IDT Technologies (Standard desalted)	AATGATACGGCGACCACCGAGATCTACACCCAACCAAACACTCTTTCCCTACACGACGCTCTTCCGATCT
P5_WTA_ AACGTTGG	IDT Technologies (Standard desalted)	AATGATACGGCGACCACCGAGATCTACACAACGTTGGACACTCTTTCCCTACACGACGCTCTTCCGATCT

(*Continued on next page*)

Continued

REAGENT or RESOURCE	SOURCE	IDENTIFIER
P5_WTA_ ACCAATCG	IDT Technologies (Standard desalted)	AATGATACGGCGACCACCGAG ATCTACACACCAATCGACACT CTTTCCCTACACGACGCT CTTCCGATCT
P7_WTA_ CCGCGGTT	IDT Technologies (Standard desalted)	CAAGCAGAAGACGGCATACGA GATCCGCGGTTGTGACTGGAG TTCAGACGTGTGCTCTTCC*G*A
P7_WTA_ TTATAACC	IDT Technologies (Standard desalted)	CAAGCAGAAGACGGCATACGA GATTTATAACCGTGACTGGAGT TCAGACGTGTGCTCTTCC*G*A
P7_WTA_ GGACTTGG	IDT Technologies (Standard desalted)	CAAGCAGAAGACGGCATACGA GATGGACTTGGGTGACTGGAG TTCAGACGTGTGCTCTTCC*G*A
P7_WTA_ AAGTCCAA	IDT Technologies (Standard desalted)	CAAGCAGAAGACGGCATACGA GATAAGTCCAAGTGACTGGAG TTCAGACGTGTGCTCTTCC*G*A
P7_WTA_ ATCCACTG	IDT Technologies (Standard desalted)	CAAGCAGAAGACGGCATACGA GATATCCACTGGTGACTGGAG TTCAGACGTGTGCTCTTCC*G*A
Software and algorithms		
Nova-ST Analysis Pipeline/Notebooks	This study	https://github.com/aertslab/Nova-ST https://doi.org/10.5281/zenodo.12608945
Carbide Create	https://carbide3d.com/	N/A
Carbide Motion	https://carbide3d.com/	N/A
bcl2fastq2 (v2.20.0)	Illumina	https://emea.support.illumina.com/downloads/bcl2fastq-conversion-software-v2-20.html RRID:SCR_015058
Sequence Analysis Viewer (v3.0)	Illumina	https://emea.support.illumina.com/sequencing/sequencing_software/sequencing_analysis_viewer_sav.html
Python (v3.8.10)	Python Software Foundation	https://www.python.org/ RRID:SCR_008394
OpenCV (v4.2.0)	OpenCV Team	https://opencv.org/RRID:SCR_015526
STARsolo (2.7.10b)	Dobin et al. (2013)[24]	https://github.com/alexdobin/STAR RRID:SCR_021542
SAW (v6.1)	BGI	https://github.com/STOmics/SAW RRID:SCR_025001
Stereopy (v0.6.0)	BGI	https://github.com/STOmics/Stereopy
Scanpy (v1.9.1)	Wolf et al. (2018)[25]	https://github.com/scverse/scanpy RRID:SCR_018139
Fiji (v1.54f)	Schindelin et al. (2019)[26]	https://github.com/fiji/fiji RRID:SCR_002285
Cell2location (v0.1.3)	Kleshchevnikov et al. (2022)[18]	https://github.com/BayraktarLab/cell2location/RRID:SCR_024859
SCope (v.1.8.2)	Davie et al. (2018)[17]	https://github.com/aertslab/SCope
Napari (v.0.4.18)	N/A	https://github.com/napari/napari RRID: SCR_022765

RESOURCE AVAILABILITY

Lead contact

Further information and requests for resources and reagents should be directed to, and will be fulfilled by the lead contact, Prof. Dr. Stein Aerts (stein.aerts@kuleuven.be).

Materials availability

This study did not generate new unique reagents.

Data and code availability

- Nova-ST and Stereo-seq data has been deposited to NCBI's GEO archive and are available as of the date of publication. Accession numbers are available in the Key Resources Table. Accessory data and information on the Nova-ST workflow can be found at: https://nova-st.aertslab.org/.
- All original code has been deposited at GitHub and Zenodo and is publicly available as of the date of publication. The Zenodo DOI is listed in the Key Resources Table.
- Any additional information required to reanalyze the data reported in this work paper is available from the Lead Contact upon request.

EXPERIMENTAL MODEL AND STUDY PARTICIPANT DETAILS

Mice were maintained in a specific pathogen-free facility under standard housing conditions (temperature 20-24°C and humidity 45–65%) with continuous access to food and water. Mice used in the study were 6–8 weeks old and were maintained on 14 h light, 10 h dark light cycle from 7 to 21 h. In this study, whole brain from male C57BL/6J was used. Animals were anesthetized with isoflurane, and decapitated. Brains were dissected and immediately snap-frozen in isopentane for 10 min. No wild animals were used in this study. Sex is not relevant for this study as we report a spatial transcriptomics technique development as main finding, therefore sex was not considered in the study design. The findings in this study apply to only one sex (male mice). All animal experiments were conducted according to the KU Leuven ethical guidelines and approved by the KU Leuven Ethical Committee for Animal Experimentation (approved protocol no. ECD P007/2021).

METHOD DETAILS

Mouse tissue preparation for cryo-sectioning

All animal experiments were performed according to the KU Leuven ethical guidelines and approved by the KU Leuven Ethical Committee for Animal Experimentation (approved protocol no. ECD P007/2021). Six to eight weeks old mice (C57BL/6J) used in the study were kept on a 14 h light, 10 h dark–light cycle from 7:00 to 21:00. Brains were dissected and immediately snap-frozen in isopentane for 10 min. Afterward, brains were embedded in Tissue-Tek OCT cryo embedding compound. Coronal cryosections (10 μm) were performed at CT = 14°C, OT = 11°C. The used area of mouse cryosections is in the somatosensory areas adjacent to the posterior parietal association areas. For each brain tissue, 5–10 OCT scrolls of 70 μm section thickness were collected into DNA lo-bind 2 mL eppendorf tubes. Ice-cold PBS was used for washing the tissue to remove the OCT matrix. Total RNA was extracted from the washed tissue sections using the innuPREP mini RNA kit (Analytik Jena; Cat. No. AJ 845-KS-2040250). The RNA quality was assessed using RNA Nano kit (Agilent). Only tissue with RIN >7 was used for the spatial analysis.

Stereo-seq optimization of tissue permeabilization

Tissue optimization was performed using the Stereo-seq Permeabilization kit (Cat. No. 111KP118) and Stereo-seq chip set P (Cat. No. 110CP118) according to the manufacturer's protocol (Stereo-seq permeabilization set user manual, Ver A1). Briefly, 4 permeabilization chips were removed from the storage buffer and washed with nuclease free water and dried at 37°C. Next, 4 consecutive 10 μm tissue sections were prepared from the tissue cryo-block and placed on the permeabilization chip, the tissue layer was thawed to attach it to the surface of the chip. After drying the tissue on a 37°C hot plate, the chip was then dipped into 100% methanol at −20°C and incubated for 30 min to fix the tissue. Post fixation, the tissue permeabilization test was performed on these chips by permeabilizing the tissue with PR enzyme prepared in 0.01N HCl (pH 2.0), at 4 different time points ranging from 6 min to 30 min. After the permeabilization, the chips were rinsed with 0.1X SSC buffer before reverse transcription. Reverse transcription was carried out at 42°C for 1 h in dark. Tissue removal was performed at 55°C for 1 h using the TR enzyme to clear the tissue before imaging. Fluorescence imaging was performed in the TRITC channel with 10X objective, following the imaging guidelines provided by the manufacturer (Guidebook for Image QC & microscope assessment and imaging, Ver A5). The optimal permeabilization time was assessed based on the strongest fluorescence signal with the lowest signal diffusion (crispness of the RNA footprint). Based on our assessment, we found the most optimal permeabilization time for the mouse brain to be 12 min.

Stereo-seq spatial transcriptomics analysis

The spatial transcriptomics analysis was performed using the Stereo-seq Transcriptomics kit (Cat. No. 111ST114) according to the manufacturer's protocol (Stereo-seq Transcriptomics set user manual, Ver A2). Briefly, as with the permeabilization analysis, the T-chip was removed from the storage buffer and washed with nuclease free water and dried at 37°C. Next, a 10 μm tissue section from a desired region of interest was prepared from the tissue cryo-block and placed on the T-chip and thawed the tissue layer to attach to the surface of the chip. After drying the tissue on 37°C hot plate, the chip was then dipped into 100% methanol at −20°C and incubated for 30 min to fix the tissue. The fixed tissue was then stained using the Qbit ssDNA reagent (Thermo Cat. No. Q10212).

Fluorescence imaging of the single-stranded DNA staining was performed in the FITC channel with a 10X objective, following the imaging guidelines provided by the manufacturer (Guidebook for Image QC & microscope assessment and imaging, Ver A5). Prior to permeabilization, the ssDNA-stained image was also subjected to QC analysis using the imageQC software as per manufacturer's recommendations. As with the permeabilization protocol, the tissue permeabilization was carried out with PR enzyme prepared in 0.01N HCl (pH 2.0) at 37°C. The optimal permeabilization time estimated from the tissue permeabilization analysis was used for the transcriptomics analysis. After washing the chip, reverse transcription mix was added to the chip and incubated at 42°C for at least 3 h. Tissue removal from the stereo seq chip was achieved by incubating the chip in the TR buffer at 55°c for 10 min cDNA release and collection was performed by incubating the chip in cDNA release mix overnight at 55°C and the released cDNA was purified with Ampure XP beads (Beckman Coulter; Cat. No. A63882) using the manufacturer's recommendation. After quality assessment using a bioanalyzer (Agilent), sequencing library preparation was performed using transposase assisted tagmentation reaction. Indexed PCR and library purification was performed to prepare the final sequencing library as per manufacturer's recommendations. Final Stereo-seq libraries were sequenced on MGI/BGI sequencing platforms and were sequenced at the MGI Latvia sequencing facility.

HDMI sequencing

The HDMI generation in this work was done using the Illumina NovaSeq 6000 S4 kit; 35 cycles (PN: 20044417). In accordance with the original publication,[10] HDMI32DraI-32 ultramer (IDT technologies – standard desalted purification) was used for first level sequencing on the NovaSeq S4 flow cell to generate the HDMI array. The HDMI32DraI-32 ultramer (details of all oligonucleotide sequences used in this work is provided in Key Resources Table) was diluted to 1 μM concentration and the actual concentration of the oligonucleotide was titrated using qPCR to estimate the final concentration to be loaded for sequencing. Briefly, we used the Kapa Library Quantification kit (Roche, KK4824) to quantify the Oligonucleotides. Based on the concentration estimated from qPCR, libraries were denatured and loaded at a final concentration of 300 p.m. on the NovaSeq 6000 following the manufacturer's instructions. Custom read primer Read1-DraI was also ordered from IDT technologies with PAGE purification.[10] The read primer was diluted to 0.3μM with HT1 buffer and loaded into the custom read primer 1 position in the NovaSeq reagent cartridge. The sequencing configuration used for reading the HDMI barcodes was 37(R1)-0(I1)-0(I2)-0(R2). At the end of the 34th cycle, the instrument was manually aborted without initiating a post run wash. The S4 flow cell was then retrieved for immediate downstream postprocessing, it can also be stored safely at 4°C for at least 2 weeks. Users not having direct access to NovaSeq 6000 instrument can instruct sequencing facility to perform the HDMI sequencing step and transport the sequenced flow cell at 4°C. Prior to shipping, the inlet and outlet ports of the flow cell should be sealed using a PDMS biopsy plugs (see the details below) to ensure the liquids in the flow channels does not dry out.

Post sequencing processing of the flow cell

Sealing of the inlet and outlet for the flow channels was achieved by plugging them with 1.5-2mm Polydimethylsiloxane (PDMS) cylinders. To prepare these cylinders, the monomer and catalyst of SYLGARD 184 Silicone Elastomer Kit (Dow chemicals) was prepared in a 10:1 weight ratio. The components were mixed thoroughly and vacuum degassed. To polymerize, the mix was poured into a 3 cm Petri dish and incubated at 80°C for 2 h to complete the polymerization process. The PDMS slab was then extracted from the Petri dish and wrapped into aluminum foil. 2 mm PDMS cylinders were prepared from this slab using a 2 mm biopsy punch (World Precision Instruments; Cat No. 504531).

The HDMI flow cells were then subjected to downstream enzymatic treatment. Firstly, the flow channels were cleaned with 200 μL of nuclease free water, NFW (Thermo Fisher; Cat No. 10977035). This was repeated for a total of three times. During each wash step, after filling the channels with reagents, a vacuum source (general vacuum pump, e.g., VWR, Cat. No. SART16694-1-60-06) was used to completely remove the reagents from the channel. Aspiration was continued until the channels became completely dry. To expose the RNA capture handle, the double stranded DNA was cut using restriction endonuclease DraI (NEB Inc. Cat. No. R0129L). The flow channels were first cleaned with 200 μL 1X rCutSmart Buffer. Then all flow channels were filled with 200 μL DraI reaction mix (1X rCutSmart Buffer, 2U/μL DraI Enzyme). Excess liquid overflowing from the outlet ports was aspirated using the vacuum pump. After making sure there were no air pockets trapped in the flow channels, the 2mm PDMS cylinder blocks were forced into the inlet and outlet ports to seal them using thin tipped forceps. The flow cell assembly was then placed into a humidification chamber (Nunc Square BioAssay Dishes; Thermo Fisher – Cat. No. 240835). For humidification, the flow cell was placed along with wet paper tissue and the Petri dish was sealed using Parafilm. The flow cell was then incubated overnight at 37°C for the completion of the endonuclease reaction.

The flow channels were washed three time with 200 μL of NFW. After the vacuum aspiration of water from the flow channels, the channels were filled with 200 μL of 1X Exonuclease buffer (NEB Inc. M0293L). After the removal of the exonuclease buffer, the flow channels were filled with 200 μL of Exonuclease reaction mix (1X Exonuclease reaction buffer, 1U/μL of Exonuclease enzyme and 0.14 U/μL of Quick Calf Intestinal Phosphatase (NEB Inc. M0525L)). After aspirating the excess reaction mix from the inlet/outlet of the sequencing flow cells, the ports were sealed with fresh 2 mm PDMS cylinders. The reaction mix was then incubated at 37°C for 45 min in the same humification chamber. After the completion of the exonuclease reaction, the HDMI flow cell assembly was retrieved, and the channels were washed three times with 200 μL of NFW. After each wash the liquid was completely withdrawn from the flow channels using a vacuum pump.

Flow cell disassembly for Nova-ST chip preparation

Manual cutting strategy

The flow cell assembly was next placed into an oven at 50°C for 20 min to dry the flow channels. The orientation of the top and bottom glass layers was identified with respect to the inlet and outlet ports of the sequencing flow cells. This is required for identifying the spatial location of the Nova-ST chips during the spatial transcriptomics analysis. The glass chip was then removed from the plastic housing by manually pulling out the plastic brackets that clamp down the inlet and outlet ports, releasing the glass chip. Then, using a fine scalpel, the gasket layer between the thin and thick glass surfaces (Figure 1D) was scored gently to separate the thin and thick glass layer. This scoring must be done carefully without damaging the functional surface of the HDMI array. After gently prying and separating the glass layers, paper masking tape was glued to the back of the glass layers (3M, Cat No. 3M 201E 48MM), excess tape was trimmed off. In this work we have used the NOMAD 3 CNC milling machine from Carbide3D to score the glass surface into a 1 cm × 1 cm cutting grid (Figure 1D). This CNC milling machine comes with a 130W spindle and has a working area of 200 × 200mm and 76mm in height. Less powerful machines can be used for the purpose described above. A diamond drag bit with a 90-degree tip, from the CNC milling machine manufacturer was used. The tip angle keeps the scoring as narrow as possible and ensures better penetration into the glass compared to a 120-degree tip. The bit is also equipped with a spring inside to adjust the force. In this case, it was adjusted so that little pressure is applied to the glass, while the tool length measurement probe of the machine can still detect the bit.

The scoring pattern was created using the machine suppliers dedicated software, Carbide Create. The glass plates dimensions were defined in the software as well as the desired scoring pattern. The scoring depths was then adapted according to the glass thickness and the direction. The thicker glass plate (1.2mm thick) was scored with a 0.6mm depth in the width direction (shortest side) and 0.2mm depth in the length direction. For the thin section (thickness 0.3mm), depths of 0.4mm and 0.1mm were used. It is important to know that the depth of cut defined in the software is not the actual depth. The actual depth differs due to the spring that retracts at the glass contact. This explains the higher depth of cut defined compared to the glass thickness. Each score was performed with a single pass of the tool.

Once the pattern was defined, the machine code (Gcode) was sent to the CNC machine via Carbide Motion, another software supplied by the manufacturer and was used to control machine movements. The plates were clamped to the table for scribing. After scoring the glass layers, the cutting was performed by using the glass running pliers (SPEEDWOX). To reduce the damage caused by the pliers on the functional surface, rubber tips were used. Before using the pliers, the pliers and the rubber tips were wiped with RNA & DNAZap followed by cleaning with 100% ethanol to ensure they were free of contamination. For cutting and preparing the Nova-ST chips, the pliers were used to cut the vertical score lines, by applying gentle pressure in the middle of the glass layers along the score line. After breaking the chips along the vertical score line, the masking tape was cut using a scalpel or razor. This was followed by breaking the glass chips along the horizontal score lines to produce the 1 cm × 1 cm Nova-ST chips. The Nova-ST chips were then pried out of the masking tape using forceps and the chips placed into 24 well plates. The location of the chip was recorded on the wells. This was repeated across the whole flow cell to produce 96 1 cm × 1 cm Nova-ST chips from both the thin and thick layers of the NovaSeq chip. In our experience breaking and preparation of the Nova-ST chips from the thick glass layer is non-trivial and sometimes getting perfect cut along the score lines is difficult. It's highly recommended to practice the cutting on trial flow cells before attempting on the HDMI flow cells. The Nova-ST chips in the 24 well plates were then subjected to following steps to remove the second strand and to store them for long term. The Nova-ST chips were washed 3X times with 0.1N NaOH. For each wash the chips were incubated in the caustic solution for 5 min, to ensure efficient denaturation of the second strand. Each of the Nova-ST chips were then washed 3X times with 1 mL nuclease free water followed by 2 times wash with 1 mL of 1X TE buffer (IDTE solution; IDT, Cat. No. 11-05-01-09). After the last wash, the chips were stored in IDT 1X TE buffer for long term storage at 4°C.

Automatic dicing strategy

After the final wash of the flow cell with NFW, the water in the flow channels was retained and the flow channels were sealed with PDMS plugs. The orientation of the top and bottom glass layers was identified with respect to the inlet and outlet ports of the sequencing flow cells, this is required for identifying the spatial location of the Nova-ST chips during the spatial transcriptomics analysis. The glass chip was then removed from the plastic housing by manually pulling out the plastic brackets that clamp down the inlet and outlet ports, releasing the glass chip. The NovaSeq flow cell was mounted on dicing tape which has a sticky backing that holds the flow cell on a thin sheet metal frame, to prepare for the dicing process. The thick glass side of the flow cell was glued on to the adhesive film. To reduce the adjustment for alignment in the dicing machine, the flow cell was glued on to the dicing tape, pre-aligned. The metal frame with flow cell was fixed to the dicing stage. The x-,y- and θ alignment was performed to align the flow cell. The origin for the start of the dicing was set. Cutting speed was set to 1 mm/s and dicing process was initiated. Firstly, the dicing was performed along the length of the NovaSeq flow cell and it was cut into 1 cm thick slabs. Without detaching the separated 1 cm slab from the dicing tape, the tape assembly was rotated by 90°, and the dicing was repeated along the width of the NovaSeq flow cell, cutting it into the desired dimensions. To ensure the glass layers of the flow cell were diced completely through, the score pattern on the dicing tape was checked. If there is no score pattern on the dicing tape, the NovaSeq chip has not been diced properly. After the dicing steps were completed, the diced NovaSeq flow cell was removed from the instrument and the dicing tape was cut out to retrieve the diced NovaSeq flow cell. With help of a sharp forceps Nova-ST chips were gently removed from the adhesive dicing tape. The Nova-ST chips at this stage still consist of two layers, the thin and thick sections still bonded by the middle gasket. A fresh razor was slid between the thin and thick sections and was used to gently pry, separating the chips. A gentle push is sufficient to separate the layers. Care should be taken to not disturb/damage the functional surface of the Nova-ST chips. Once the layers have been

separated, the separated chips were placed on a paper towel with the functional surface facing up. After separating a batch of chips, using a sharp forceps, the chips were transferred to the respective 24 well plates, with the functional surface of the chips facing upwards. The Nova-ST chips in the 24 well plates were then subjected to following steps to remove the second strand and to prepare them for long term storage. The Nova-ST chips were washed 3X times with 0.1N NaOH. For each wash the chips were incubated in the caustic solution for 5 min, to ensure efficient denaturation of the second strand. Each of the Nova-ST chips were then washed 3X times with 1 mL nuclease free water followed by 2 times wash with 1 mL of 1X TE buffer (IDTE solution; IDT, Cat. No. 11-05-01-09). After the last wash, the chips were stored in IDT 1X TE buffer for long term storage at 4°C.

RNA quality assessment

Prior to the tissue optimization and spatial transcriptome analysis on the Nova-ST chips, every tissue analyzed in this work was subject to RNA quality assessment. In brief, 5–10 serial sections of 50–70 μm thickness were cryo-sectioned from a region farther away from the region of interest. These serial sectioned OCT scrolls were put into a 2 mL lo-bind tube (Eppendorf Cat. No. 0030108078) and stored at −80°C. The tissue scrolls were washed with 1 mL of ice-cold PBS at 4°C. Total RNA extraction from the spun-out tissue was performed using innuPREP mini-RNA kit (Analytik Jen; Cat. No. AJ 845-KS-2040250). Manufacturer recommendations were followed to extract total RNA. Elution was performed in 30 μL of NFW. The quality of the total RNA was assessed using Pico RNA kit (Agilent).

Optimization of tissue permeabilization for Nova-ST workflow

Optimal tissue permeabilization for mouse brain samples analyzed in this work was estimated using the 10X Visium Spatial Optimization kit (PN 1000192). Briefly, serial sections of the tissue were sectioned from the OCT embedded tissue matrix and placed on the capture spots of Visium Spatial Tissue Optimization slide (10X Genomics, PN: 3000394). To estimate the optimal permeabilization time, pepsin (Sigma Aldrich; Cat. No. P7000) at a concentration of 1 mg/mL in 0.1N HCl (Fisher Scientific Cat. No. AA35644K2) was used with different incubation times (5, 10, 15, 20, 25, 30, 35 min). The rest of the protocol was followed as per the manufacturer's recommendations (10X Genomics; Visium Spatial Gene Expression Reagent Kits – Tissue Optimization; CG000238 Rev E) to determine the most optimal time for tissue permeabilization. Imaging was performed using a Nikon NiE upright microscope equipped with a 10x Plan Apo lambda 0.45 air lens and a black and white sCMOS camera Prime BSI (Teledyne Photometrics). The setup was controlled by NIS-Elements (5.42.04, Nikon Instruments Europe B.V.). TRITC was excited with 550nm (CoolLED pE-800) and collected with a 577-630nm emission filter. A large tilescan was acquired to cover the entire tissue and chip area using 10% overlap and a focus surface. Like Stereo-seq, the optimal permeabilization time was assessed based on the strongest fluorescence signal with the lowest signal diffusion (crispness of the RNA footprint). Based on our assessment, we found the permeabilization time of 27 min optimal for mouse brain sections.

Nova-ST workflow: Tissue preparation, permeabilization & first strand synthesis

As with Stereo-seq experiments, the OCT embedded tissues were sectioned to a thickness of 10μm, placed on the capture area of the Nova-ST chip and melted. If the samples were not immediately processed for transcriptome capture, the Nova-ST chip was refrozen on cryoblock and stored in −80°C and in our experience the quality of the tissue is not impacted with 2–3 weeks of storage of the tissue section at −80°C.

Standard Hematoxylin and Eosin (H&E) staining procedure was used to stain the tissue. Briefly, the Nova-ST chip with frozen tissue section was taken from −80°C storage and immediately melted on a 37°C block for 1 min. The tissue was then fixed in methanol at −20°C for 30 min. Post fixation, the tissue was dehydrated by adding 150μL of isopropyl alcohol (IPA) and incubating for 1 min. After removal of the IPA, the Nova-ST chip was air dried for 3 min (or until the chip is completely dried). 200 μL Mayer's haematoxylin (Agilent, Cat. No. S3309) was added to the chip and incubated for 7 min. Using a forceps the chip was washed in excess NFW. 150μL of bluing buffer (Agilent, Cat. No. CS702) was added to Nova-ST chip and incubated for 2 min. The chip was again washed with excess NFW. The tissue was then treated with 200 μL of Eosin-Y buffered solution (10% v/v of Eosin-Y (Sigma, Cat. No. HT110216) in 0.45 M Tris Acetic acid solution pH 6.0) and incubated for 1 min. The chip was dried at 37°C for 5 min (or until the water was completely evaporated) prior to imaging. Brightfield imaging was performed using a Nikon NiE upright microscope equipped with a 10x Plan Apo lambda 0.45 air lens and a color camera DFK 33UX264 (The Imaging Source, LLC). The setup was controlled by NIS-Elements (5.42.04, Nikon Instruments Europe B.V.). A large tile scan was acquired to cover the entire tissue and chip area using 10% overlap and a focus surface. Post imaging, the sample was immediately processed for the spatial transcriptomics workflow.

Pepsin reagent prepared in 0.01N HCl (pH 2.0) (1 mg/mL) was preheated in a 37°C oven. After H&E staining and imaging, using forceps, the Nova-ST chip was placed into a 3 cm Petri dish. 300μL of prewarmed pepsin was added to the H&E stained tissue and permeabilization was performed at 37°C for the optimal permeabilization time estimated in the previous step. After the permeabilization step, the pepsin solution was blotted off from the Nova-ST chip and the chip was transferred into a 24 well plate. The permeabilization reaction was stopped by sequentially washing the chip with 0.1X SSC (20X SSC; Thermo Fisher; Cat. No: 15557044), followed by 300 μL of 1X RT wash buffer (1X Maxima h- Reverse Transcriptase buffer; Cat. No EP0753, 1U/μL Lucingen NxGen RNase Inhibitor; Cat. No. 30281-2). Finally, 300 μL of First Strand mix was added to the well (1X Maxima h- Reverse Transcriptase buffer, 1U/μL Lucingen NxGen RNase Inhibitor, 4% Ficoll PM-400; Sigma Aldrich Cat. No. F4375-10G, 1 mM dNTP; Thermo Fisher Cat. No. R1121, 10U/μL Maxima RTase). The wells were covered with multiple layers of square patches of Parafilm. The 24 well plate was then sealed and put into oven at 42°C for overnight incubation for first strand synthesis.

Nova-ST workflow: Exonuclease treatment

In this step, exonuclease treatment was performed on the Nova-ST chip to remove single stranded HDMI capture tags without the first strand product to avoid the undesired secondary downstream reactions. After the first strand reaction, the FSS mix was removed

from and the Nova-ST chip and was washed with 300 μL of 0.1X SSC. The chip was washed with 300 μL of 1X Exonuclease I buffer before adding 300 μL Exonuclease reaction mix (1X Exonuclease reaction buffer, 1U/μL of Exonuclease enzyme). The reaction was incubated at 37°C for 45 min.

Nova-ST workflow: Tissue clearance

After incubation, the tissue on the surface of the Nova-ST chips was cleared. In this step, the exonuclease reaction mix was removed from the well and 300 μL of Tissue clearance reagent was added to the well containing the Nova-ST chip (100 mM of Tris pH 8.0; Thermo Fisher Cat. No AM9856, 200 mM of NaCl; Thermo Fisher Cat. No. AM9760G, 2% SDS; Thermo Fisher Cat. No. 24730020, 5 mM EDTA; Thermo Fisher Cat. No. 15575020, 16U/μL Proteinase K; NEB Inc Cat. No. P8107S). The reaction was incubated at 37°C for 45 min to complete the tissue removal reaction.

Nova-ST workflow: Second strand synthesis

Before the subsequent processing, clearance of the tissue was ensured from the surface of the Nova-ST chip. Then the Nova-ST chip was washed three times with 3 mL of NFW. The chip was them washed three times with 0.1N NaOH. During each wash, the chip was incubated in 0.1N NaOH for 5 min to remove the mRNA strand. The Nova-ST chip was then washed three times with 0.1M Tris-HCl (pH 7.5) (Thermo Fisher; Cat. No. 15567027) followed by 3X times wash with NFW. Using forceps, the chip was transferred to another well in the 24 well plate. Before transferring excess water was blotted off from the bottom of the chip surface. The chip was then subjected to second strand synthesis reaction. 300μL of Second strand synthesis reaction mix (1X NEB Buffer 2, 10 mM RPE randomer, 1 mM dNTP, 0.5 U/μL Klenow Fragment (3'→5′ exo-); NEB Inc Cat. No. M0212L) was added to the well. The chip was incubated in the second strand reaction mix for 2 h at 37°C.

Nova-ST workflow: Second strand product extraction, cleanup and random primer extension PCR

The Nova-ST flow cell was washed three times with 3 mL of NFW. Using forceps, the chip was transferred from the well to a 3 cm Petri dish. Before transferring excess water was blotted off from the bottom of the chip surface. 90μL of 0.1 M NaOH was added to the surface of the Nova-ST chip, it was then incubated on the chip for 5 min. After the incubation, the liquid was harvested into a DNA lo-bind 1.5 mL eppendorf tube. This process was repeated two additional times. After the final collection, the volume of the RPE collect was estimated and 0.28 times the volume of Tris 7.0 (Thermo Fisher Cat. No. AM9851) was added to the RPE collection to neutralize the reaction. After 2 min of incubation, the RPE product was purified using 1.8X Ampure XP beads as per manufacturer's recommendation. The magnetic bead elution was performed with 44 μL of EB buffer (Qiagen Cat. No. 19086). 42 μL of elute was taken for the RPE PCR. PCR was performed on purified RPE product by adding the PCR mix (1X KAPA HiFi master mix; Roche Cat. No. KK2602, 1 μM RPE forward primer, 1 μM RPE reverse primer). The following RPE PCR program was used for the product amplification: 95°C–3 min, 14 cycles of {95°C–30s; 60°C–1 min; 72°C–1 min}, final extension of 72°C – 5mins. The PCR product was then purified with 0.8X Ampure XP beads with elution in 40 μL of EB buffer.

Nova-ST workflow: Index PCR and sequencing

The RPE amplified library was quantified using the Qubit dsDNA Quantification kit (Thermo Fisher Cat. No. Q32851) and the size distribution of the RPE amplified library was estimated using High Sensitivity DNA kit (Agilent). Based on the measurements 10 nM RPE library dilution was performed using NFW. Final indexing PCR was performed by adding the PCR mix (1X KAPA HiFi master mix, 1 μM WTA forward primer, 1 μM WTA reverse primer and 2nM of RPE library). The following WTA PCR program was used for the product amplification: 95°C–3 min, 14 cycles of {95°C–30s; 60°C–30 s; 72°C–30 s}, final extension of 72°C – 5mins. Two rounds of purification were performed on the amplified PCR product with 0.8X Ampure XP beads and the final elution in 60 μL of EB buffer. Sequencing of the Final Nova-ST libraries was performed on NextSeq 2000 sequencing platform. The concentration of the libraries were normalized to 2 nM using RSB buffer with Tween 20 (Illumina; Cat. No. 20512944). The 2 nM library was further diluted to 800 p.m. before loading to the instrument. The libraries were sequenced with the following sequencing specification: R1 = 34 bps; I1 = 8 bps; I2 = 8bps; R2 = 91 bps.

Data analysis – HDMIs

Raw sequencing data was loaded into Illumina's Sequence Analysis Viewer to first, broadly check that the base composition matches the expected sequence (Figure S2) and secondly, to identify any tiles which were not sequenced – these were noted for exclusion in later steps. Raw data was next demultiplexed using Illumina's bcl2fastq (v2.20.0), creating one set of fastq files per tile of the flow cell using the following command: bcl2fastq -R ${RUN_FOLDER} -o Demultiplexed/${TILE_NO} -r 1 -p 1 -w 1 –tiles s_<${TILE_NO}> –use-bases-mask = y32n* –minimum-trimmed-read-length = 32 –write-fastq-reverse-complement. For each read in each fastq file, the barcode sequence was first checked against the expected degenerate sequence and then the tile number, X coordinate, Y coordinate and read sequence, were recorded and saved to disk. A small subset (10,000) of valid reads was saved to a separate file for later use.

As the X and Y coordinates obtained during the previous step were local coordinates and specific to the tile that each read came from, to properly reconstruct the spatial location of every read from the chip, these coordinates were put within a global context, we use the fiducial markers (concentric circles) present within each tile to align them to each other. This step can either be performed on a per-chip basis, or across all tiles from the HDMI generation, but was performed per chip for the data in this study. To do this, for each tile processed, a numpy array (max_x, max_y) was created and the value at each coordinate where a valid barcode is found was set to 1. These matrices were trimmed and reshaped to bin the data in 25x25 bins, simplifying processing. Matrices were normalized to a maximum of 255 and then converted to greyscale images using OpenCV (v4.6.0). These images were inverted, denoised (cv.fastNlMeansDenoising, h = 100) and thresholded (min 128, max 255) to extract and image of just the fiducial markers. Next,

Hough circle detection was used at 2 different radii (1. minRadius = 40,maxRadius = 80, 2. minRadius = 15,maxRadius = 30) to detect the coordinates of the inner and outer fiducial circles within each tile and the centroid of each set was calculated when all (8) circles were identified successfully. Where all 8 fiducials were not identified, centroid coordinates were interpolated using the adjacent tiles. Distances between fiducial circles were measured in the H&E and electron microscopy images (in both rows/swaths and columns) and these distances were used to calibrate the scale of the coordinates extracted from the fastq files to nm. HDMI coordinates were then corrected per tile to place the fiducial circles the correct distance from each other in both directions, beginning with the top left tile of a Nova-ST chip. Swaths 1, 3 and 5 begin at the same position, swaths 2, 4 and 6 are offset by a single tile, and this was accounted for in this correction.

Data pre-processing – Spatial libraries

Barcodes from the first 1 million reads from read 1 of the RNA sequencing were extracted and compared with the subset taken in HDMI Basic Processing to identify the tiles in the Nova-ST chip used which have reads (i.e., were under the tissue section). All barcodes from the HDMI data of the tiles covered by the section were then extracted into a whitelist for STARsolo (2.7.10b), these were trimmed to 31bp to allow STARsolo to perform a Hamming error-correction. For each read of the spatial library, the UMIs present in read 2 (the first 9 base pairs) were extracted and appended to the corresponding read from read 1 and STARsolo was run with the following parameters: –soloType CB_UMI_Simple –soloCBwhitelist ${BARCODE_WHITELIST_FILE} –soloCBstart 1 –soloCBlen 31 –soloUMIstart 32 –soloUMIlen 8 –soloBarcodeMate 0 –soloBarcodeReadLength 0 –soloFeatures Gene GeneFull –soloCBmatchWLtype 1MM –soloUMIdedup 1MM_All –soloCellFilter None –outSAMtype BAM SortedByCoordinate –outSAMattributes NH HI AS nM CR CY UR UY CB UB sS –readFilesIn ${READ_2} ${READ_1_PLUS_UMI}

Matrices from STARsolo and the corrected coordinates were combined into a GEM file, a format used by BGI's Stereo-seq pipeline, to enable the loading and binning functions of Stereopy, as well as consistent analysis of the two data types.

Data analysis – Spatial libraries

Stereo-seq matrices were loaded into Python using Stereopy (v0.6) at three different bin sizes, 50, 100 and 200, for Nova-ST, the following sizes were used 728, 1456, 2912, where each size corresponds to the same dimensions as the Stereo-seq bins. Following data loading, samples were further analyzed using Scanpy, in brief: Standard quality control metrics were calculated and bins with too few genes detected were removed (Bin 50/728: 75 Genes, Bin 100/1456: 250 Genes, Bin 200/2912: 500 Genes), bins were not filtered by mitochondrial percentage. Bins were normalized to a total of 10,000 counts and log transformed before highly variable genes were identified. A regression for total counts and mitochondrial percentage was applied and counts were scaled to unit variance with a mean of 0, counts above 10 were clipped to 10. A principal component analysis was performed, followed by neighborhood detection, UMAP generation and Leiden clustering at several resolutions. H&E images were aligned to spatial data using the BigWarp Fiji plugin, selecting multiple fiducial circles visible in both the H&E and spatial data as landmarks and using an affine transformation.

For cell2location,[18] the single cell mouse cortex and hippocampus data from Yao et al. 2021[19] was loaded from the provided h5 files into an Anndata object and associated metadata was added. This object was then subsampled without replacement to contain half of the original cells to simplify training the cell2location model. A cell2location RegressionModel instance was created using the single cell data with external_donor_name_label as the batch key and subclass_label at the labels key, and the model was trained with 500 max epochs. The cell abundance estimations were exported using the following parameters: num_samples = 1000, batch_size = 2500. Next, both the spatial and single cell data were subset to only include genes present in both datasets and a Cell2location model was set up using the spatial data (at bin50), the exported reference cell states and N_cells_per_location = 2. This model was then trained using the following parameters: max_epochs = 30000, batch_size = None, train_size = 1) and the final estimated cell abundances was exported with num_samples = 1000 and batch_size equal to 1/10th of the dataset size. Anndata objects were converted into loom files, including the spatial coordinates and cell2location prediction scores for visualization in SCope.

Data analysis - Method comparison

Nova-ST results were compared to other methods that profiled the mouse brain: Visium-HD (Downloaded from https://www.10xgenomics.com/datasets/visium-hd-cytassist-gene-expression-libraries-of-mouse-brain-he), Stereo-seq in house generated dataset, Stereo-seq Chen et al. 2022 Cell,[8] Stereo-seq You et al. 2023 bioRxiv,[20] osmFISH Codeluppi et al. 2018 Nature Methods.[21] Where possible, datasets were chosen to contain the somatosensory cortex. However, the Chen et al. dataset was located more posterior in the brain. To compare the sensitivity, data from a corresponding area, comprising the cortex, corpus callosum and cornu Ammonis 1 (CA1), was selected manually using the Napari viewer. The gene counts were normalized to the measured tissue area and compared to Nova-ST. To estimate the transcript capture efficiency, the counts per area were compared to high sensitivity osmFISH counts of the same brain area.[21]

QUANTIFICATION AND STATISTICAL ANALYSIS

All analyses were performed using Python 3.8.10 unless otherwise mentioned. Pearson correlations were used to compare sample similarities in Figure S4. All other packages as well as their version and any parameters used are detailed within the STAR Methods.

Cell Reports Methods, Volume *4*

Supplemental information

Nova-ST: Nano-patterned ultra-dense platform for spatial transcriptomics

Suresh Poovathingal, Kristofer Davie, Lars E. Borm, Roel Vandepoel, Nicolas Poulvellarie, Annelien Verfaillie, Nikky Corthout, and Stein Aerts

Supplementary Figures:

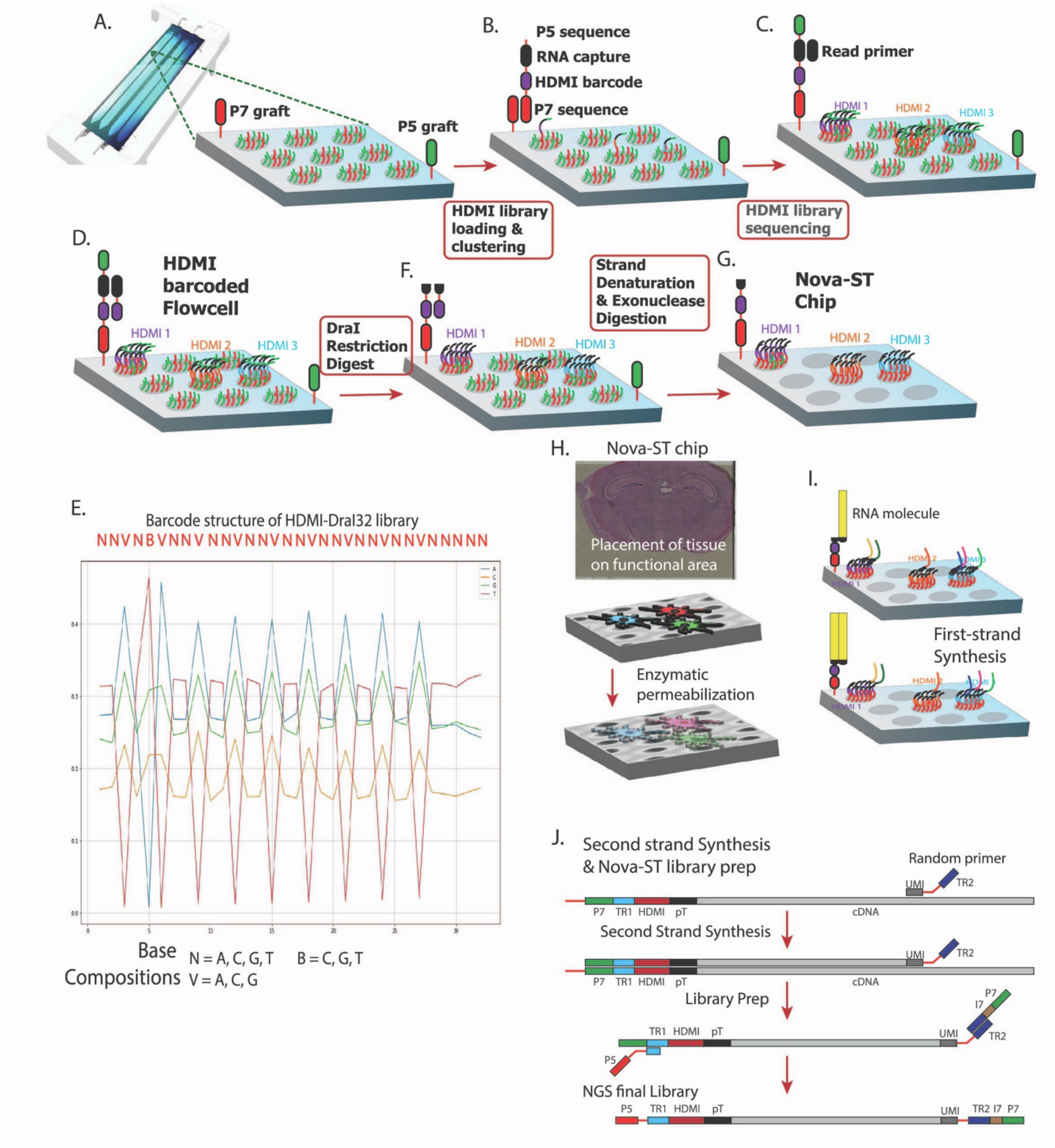

Figure S1: Summary of Nova-ST workflow. Related to Figure 1, 2 & STAR Methods section.
A.) Cartoon depicting nanowells with the functionalized sequencing handles in the NovaSeq 6000 S4 flow cell.
B-D.) HDMI sequencing workflow where the HDMI oligonucleotide (HDMI-DraI 32) undergoes annealing with the sequencing handles in the nanowell followed by isothermal bridge amplification, in which several thousand copies of the HDMI strand are created. During sequencing, a custom read primer (Read1-DraI primer), reads the HDMI spatial barcode in the backbone of the HDMI oligonucleotide sequence.

E.) An example of base composition of the sequenced HDMI library compared to the actual sequence of the HDMI spatial barcodes in the HDMI-DraI32 ultramer.
F.) Post sequencing, the Dra-I restriction enzyme cuts the double strand sequence at the TTTAAA locus to free the capture domain.
G.) The double stranded DNA is denatured using caustic treatment to expose the RNA capture domain for spatial transcriptomics.
H.) Tissue overlayed on the functional surface of the Nova-ST chip. Diagrammatic description of the enzymatic digestion of tissue on the surface of the Nova-ST chip.
I.) mRNA captured from the permeabilized tissue followed by the first strand synthesis on the surface of the Nova-ST chip.
J.) Downstream processing of the spatial library after the removal of the tissue from surface of Nova-ST chip, where second strand synthesis is achieved by a random primer extension (RPE). The extended second strand product is denatured and the rest of the RPE product amplification and the indexed NGS library preparation is performed in a tube.

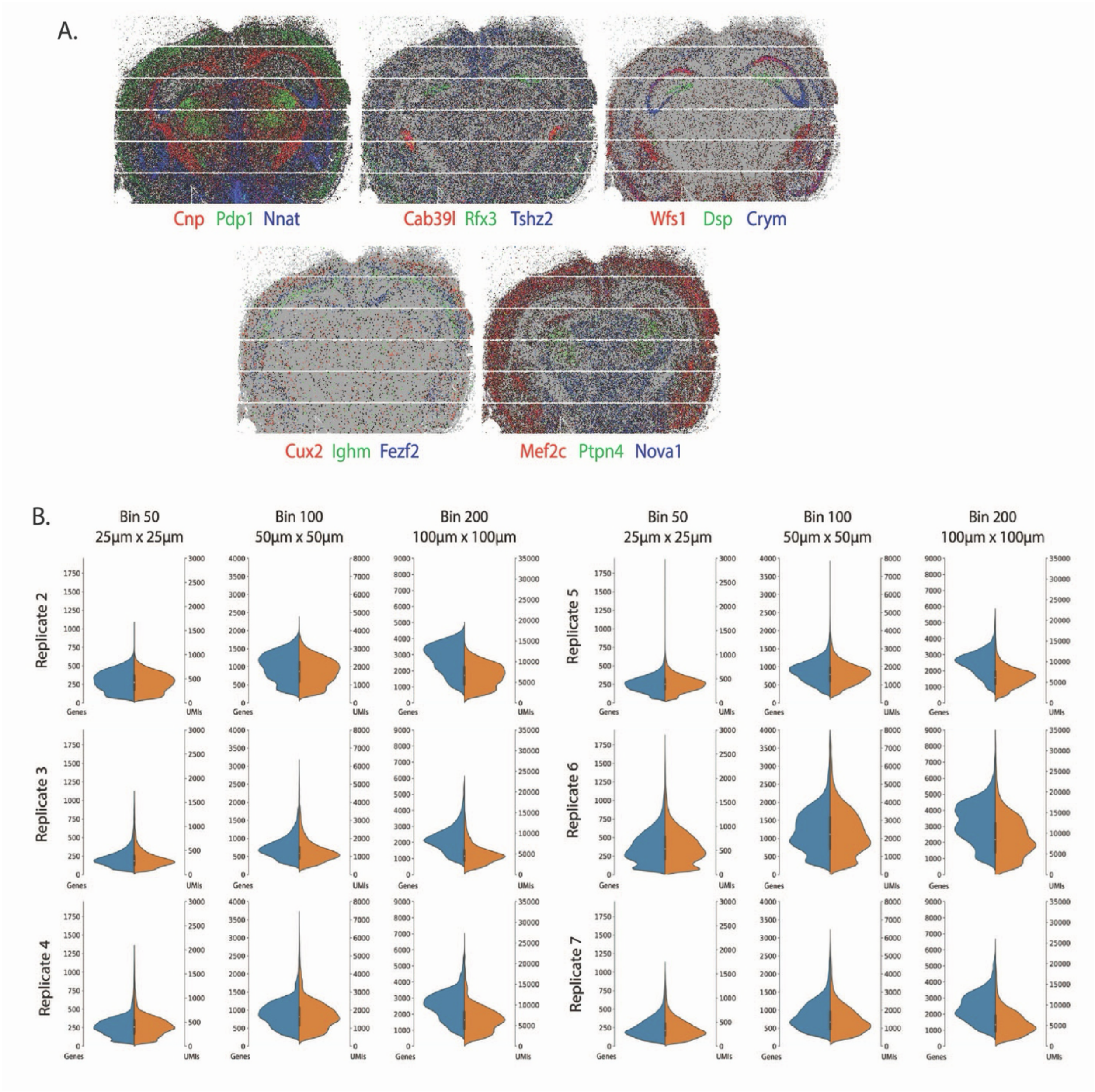

Figure S2: Quality comparisons of Nova-ST samples showing spatial localizations and general sequencing statistics. Related to Figure 3.

A.) Various sets of spatially expressed genes displaying specific patterns throughout the brain including specificity in the cortex and hippocampus.

B.) Gene (blue) and UMI (orange) distributions for all shallowly sequenced replicates at all three bin sizes (50, 100 and 200).

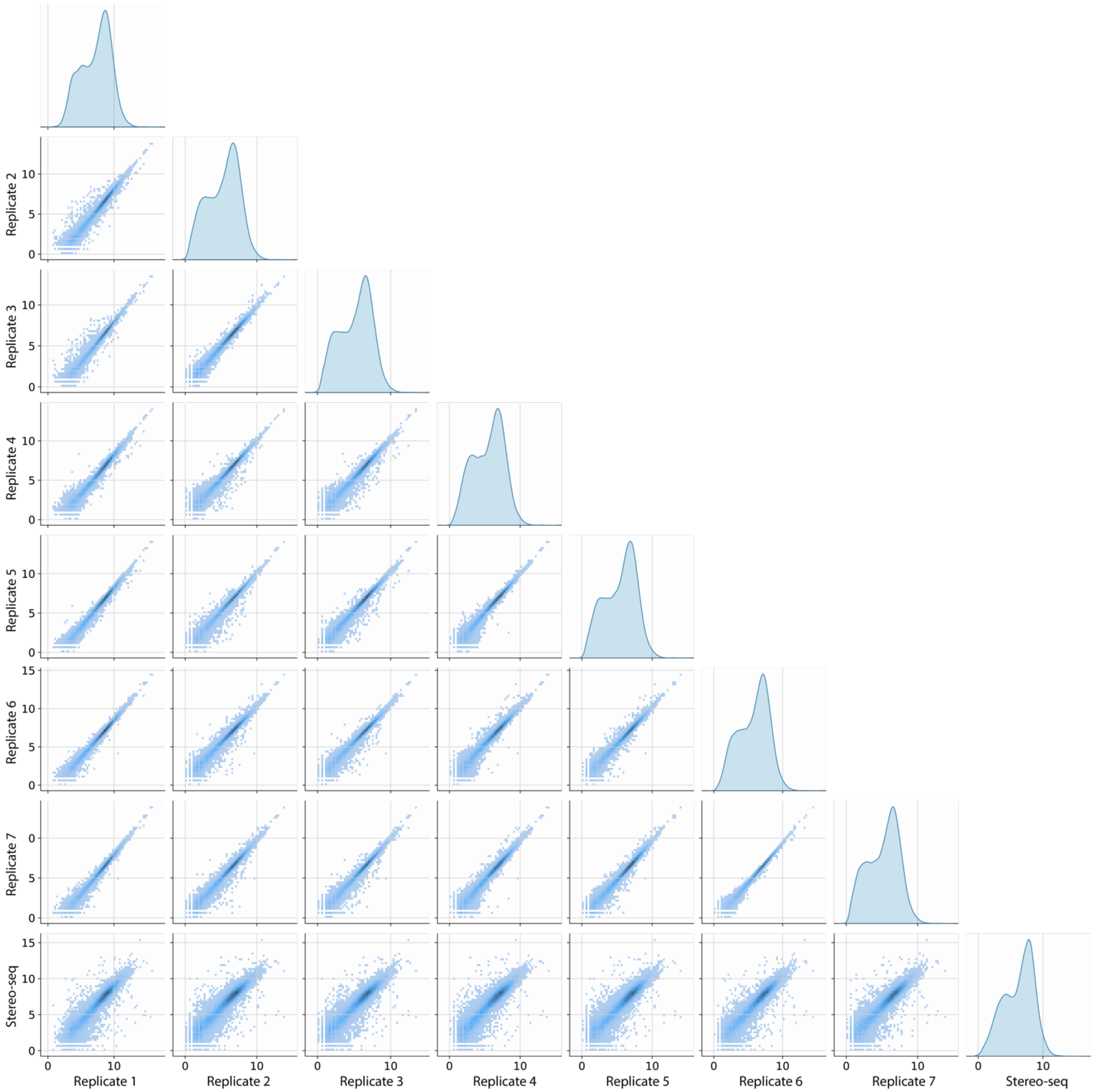

Figure S3: Correlation analysis with all replicates in Nova-ST trials. Related to Figure 3.
Pairwise comparisons of summed gene counts within each sample, log-log axes. Darker areas indicate higher genes density. Self-comparisons are excluded, only genes found in all samples are included.

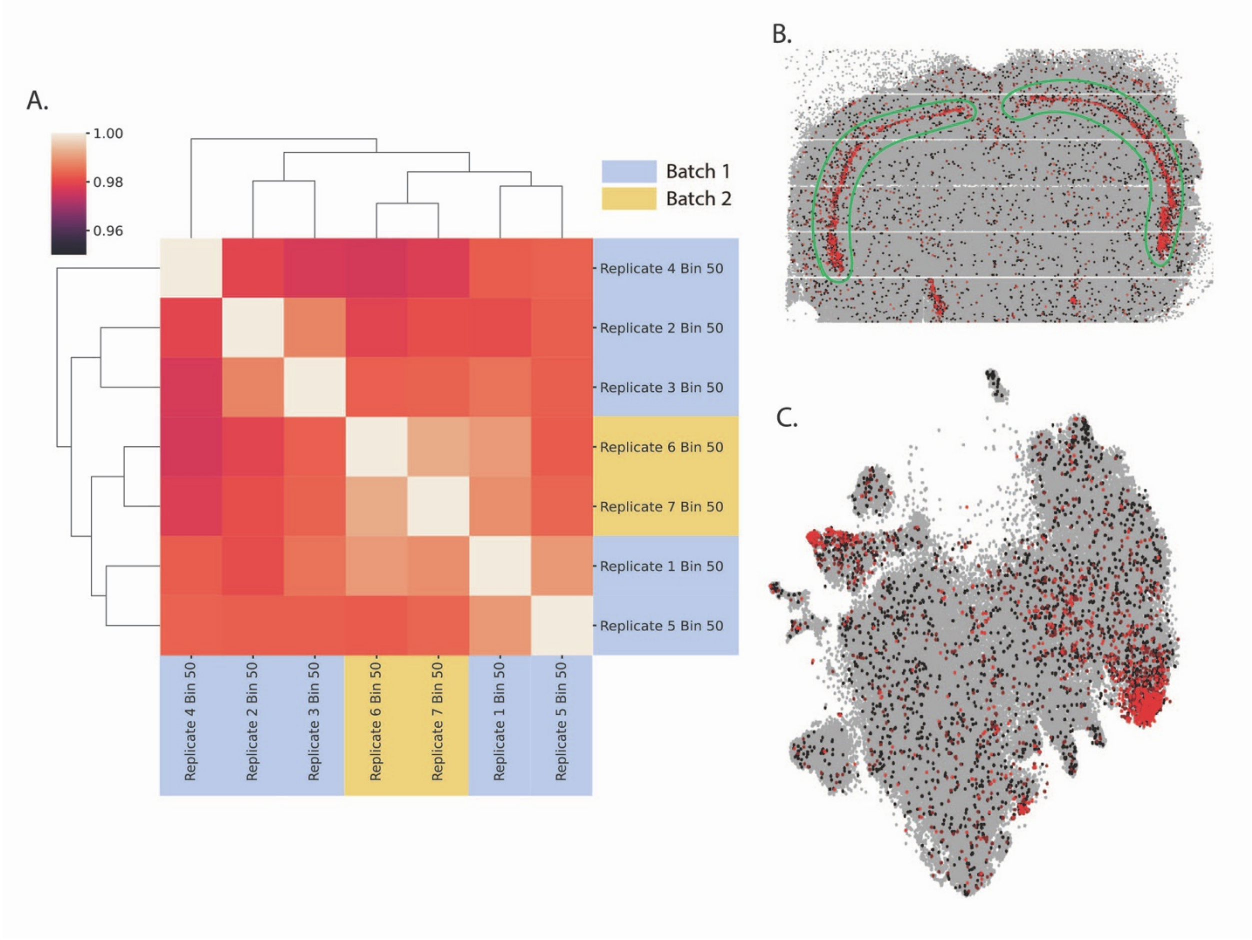

Figure S4: Summed genes correlation analysis with replicates in Nova-ST trials and diffusion of transcripts with Nova-ST. Related to Figure 3.

A.) Pairwise Pearson correlation values of summed gene counts (log scale) between each of the replicate samples. Hierarchical clustering shows no batch effect between different batches of Nova-ST chips sample.

B-C.) Spatial visualizations and t-SNE for Nova-ST DS data. Expression of Ccn2 is localized to the cortex layer 6b and corresponding location of these bins in t-SNE is highlighted by the green contours.

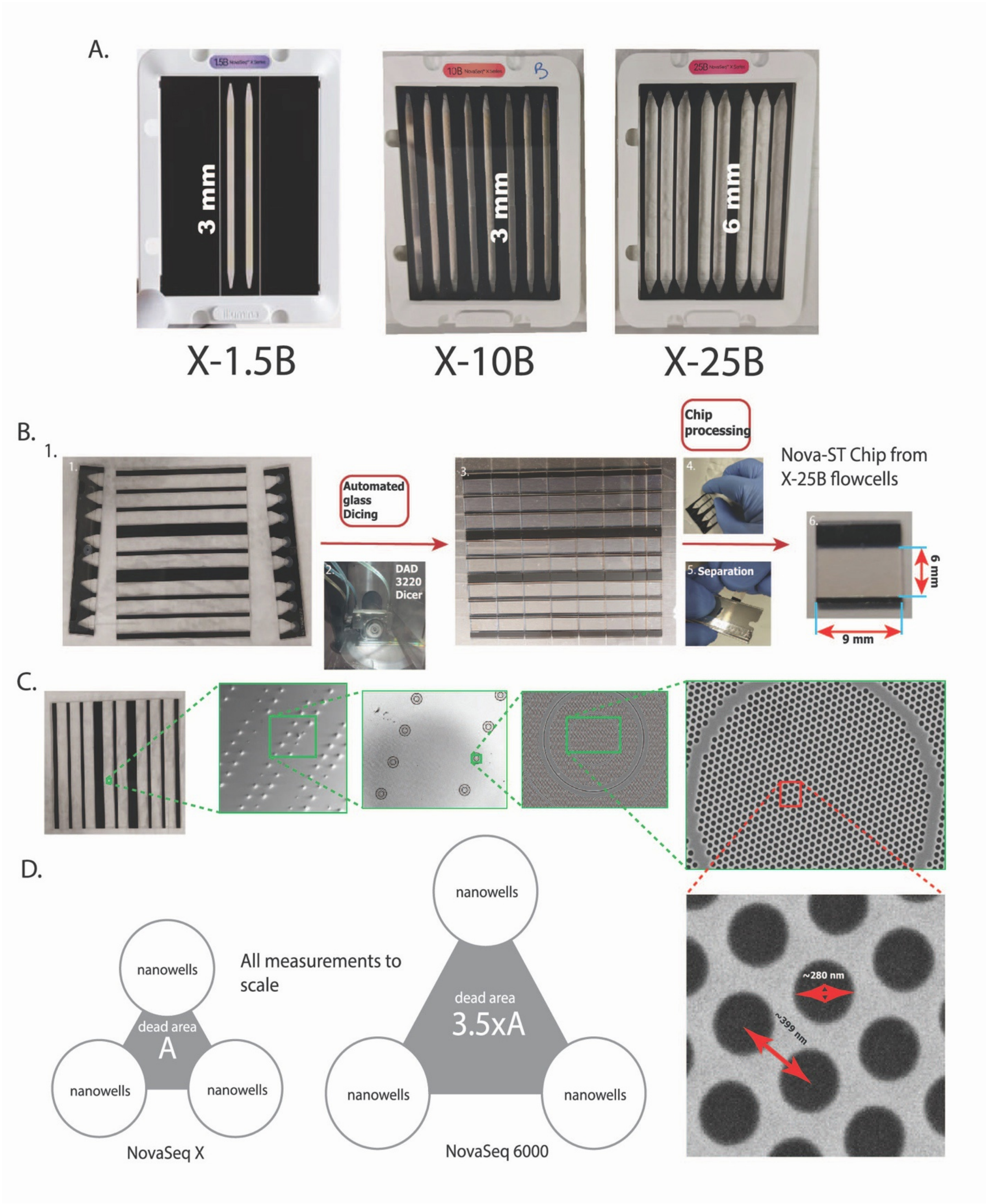

Figure S5: NovaSeq-X processing details for preparing Nova-ST chips. Related to Figure 1 & STAR Methods.

A.) Different flow cell variants of the NovaSeq X sequencer, with the thickness of the flow channel (active area) indicated.

B.) Details of automated cutting: 1. A grid pattern of chip size is created, along which the flow cells are cut to prepare the Nova-ST chips. 2. Cutting the flow cell into chips using the DAD 3220 Dicer. 3. The diced flow cell, still attached

to a dicing tape. 4. Removal of the chips from the backing. 5. Separating the chip layers using a sharp razor blade 6. Final Nova-ST chips derived from NovaSeq-X flowcell.

C.) Electron micrograph characterization of the functional surface of the X-25B flow cell. Zoomed-in pictures of different features on the functional surface of the flow cell and the dimensions and the pitch separation of nanowells arranged in a hexagonal grid pattern.

D.) Pictorial description of a scaled comparison of the dead space area between the nanowells in the NovaSeq X and 6000 flow cells.

Supplementary Tables

Table S1: Sequencing statistics related to the different replicates of Nova-ST replicates. Related to Figure 3.

	total_reads	valid_hdmis	invalid_hdmis	discarded_hdmis	relevant	not_relevant
Replicate_1	1229694204	815856409	413837043	0	815856409	0
Replicate_2	76787319	50444015	26342673	0	50444015	0
Replicate_3	89431881	56140800	33289381	0	56140800	0
Replicate_4	111141907	71832838	39308771	0	71832838	0
Replicate_5	97427512	63896606	33530653	0	63896606	0
Replicate_6	139751725	94143058	45607905	0	94143058	0
Replicate_7	79238612	51419396	27818647	0	51419396	0
BGI_Mouse_Brain	1229691448	977259390	252432058	10742731	861023071	116236319

	mapped	unmapped	multimap	annotated	unannotated	dedup_umis
Replicate_1	667007549	122433935	25961455	5.71E+08	95759874	208395033
Replicate_2	42562771	6021148	1757650	37920155	4642616	29806967
Replicate_3	45662443	8366885	1970136	39587409	6075034	25218192
Replicate_4	61137185	8304585	2236574	53594687	7542498	34405898
Replicate_5	54293446	7344408	2121376	47846949	6446497	34647069
Replicate_6	74001874	17058971	2876680	61874500	12127374	46641749
Replicate_7	39780826	9906065	1555548	33864218	5916608	25640370
BGI_Mouse_Brain	671363777	53511081	136148213	5.39E+08	132832578	84854065

	dup_umis
Replicate_1	362852642
Replicate_2	8113188
Replicate_3	14369217
Replicate_4	19188789
Replicate_5	13199880
Replicate_6	15232751
Replicate_7	8223848
BGI_Mouse_Brain	453677134
